# Drawn-on-skin electronic tattoo as a closed-loop sensing-stimulation system for the muscles

**DOI:** 10.1126/sciadv.aed7673

**Published:** 2026-04-15

**Authors:** Ya Huang, Zhenlin Chen, Jingkun Zhou, Huiling Jia, Lung Chow, Yu Zhou, Shengxin Jia, Binbin Zhang, Faheem Ershad, Shubham Patel, Chun Ki Yiu, Yuyu Gao, Qiang Zhang, Xingcan Huang, Jian Li, Kuanming Yao, Guangyao Zhao, Peining Chen, Huisheng Peng, Dong Sun, Cunjiang Yu, Xinge Yu

**Affiliations:** ^1^ State Key Laboratory of Molecular Engineering of Polymers, Department of Macromolecular Science, Institute of Fiber Materials and Devices, and Laboratory of Advanced Materials, Fudan University, Shanghai, China.; ^2^ Department of Biomedical Engineering, City University of Hong Kong, Kowloon, Hong Kong, China.; ^3^ Hong Kong Center for Cerebra-Cardiovascular Health Engineering, Hong Kong Science Park, New Territories, Hong Kong, China.; ^4^ College of Future Information Technology, Fudan University, Shanghai, China.; ^5^ Department of Electrical and Computer Engineering, University of Illinois Urbana-Champaign, Urbana, IL 61801, USA.; ^6^ Department of Electrical and Computer Engineering, University of Houston, TX 77204, USA.; ^7^ Materials Research Laboratory, University of Illinois Urbana-Champaign, Urbana, IL 61801, USA.; ^8^ Department of Engineering Science and Mechanics, Pennsylvania State University, University Park, PA 16802, USA.; ^9^ Institute of Digital Medicine, City University of Hong Kong, Kowloon, Hong Kong, China.; ^10^ Department of Materials Science and Engineering, Department of Bioengineering, Department of Mechanical Science and Engineering, Nick Holonyak Micro and Nanotechnology Laboratory, Beckman Institute for Advanced Science and Technology, University of Illinois Urbana-Champaign, Urbana, IL 61801, USA.; ^11^ City University of Hong Kong Shenzhen Research Institute, City University of Hong Kong Shenzhen, Shanghai, China.

## Abstract

Simultaneous electromyography (EMG) sensing and closed-loop electrical stimulation (ES) could enable interactive muscle training, motor rehabilitation, and kinesthetic feedback. However, current systems often suffer from nonconformal device/skin interfaces, poor wearability, and anatomical variability among users, limiting signal fidelity and stimulation precision. Here, we present a fully wireless, skin-integrated electronic tattoo platform that records high-quality EMG and delivers closed-loop ES through custom drawn-on-skin, conformal electrodes with strong adhesion and high spatial accuracy. The system supports closed-loop muscle interactions across body regions and even between individuals. Machine learning models classify EMG patterns from different hand gestures with >90% accuracy and adapt ES parameters to stimulate specific muscle groups. In a heavy object holding task, users reached the required grip behavior substantially faster than unassisted controls, indicating improved neuromuscular efficiency. This closed-loop framework not only supports personalized muscle control but also supports coordinated activation across multiple sites or users, unlocking possibilities in interactive motor training, remote rehabilitation, and virtual reality environments.

## Teaser

Electronic tattoos can precisely sense and stimulate target muscles, enabling advanced rehabilitation and motor control.

## INTRODUCTION

Electrical stimulation (ES) is a typically used technique that delivers electrical currents through the skin to activate nerves, thereby aiding movement or providing feedback in individuals (*1*, *2*). ES plays a critical role in rehabilitation, which seeks to restore physical, sensory, and cognitive functions impaired by injury, illness, or disability (*2*, *3*). Among these functions, motor recovery, in particular the restoration of fine motor control, is essential and typically relies on therapeutic interventions such as physical therapy and neuromodulation. However, traditional ES approaches often use generalized treatment protocols, which may not account for individual variability in anatomy, physiology, or pathology. This lack of personalization can result in suboptimal outcomes, including prolonged recovery times or incomplete functional control (*4*, *5*). The limitations are especially pronounced for fine motor control, such as hand gestures, which depend on the precise coordination of multiple muscle groups in the forearm. Restoring these complex movements requires target and adaptive neuromodulation strategies.

Recent advances in real-time muscle monitoring and targeted muscle activation have opened promising avenues for enhancing the personalized, adaptive, and precise treatment (*5*, *6*). These technologies not only enhance rehabilitation outcomes but also enable kinesthetic feedback, expanding their utility to fields such as remote control systems (*7*) and virtual reality assisted rehabilitation (*8*, *9*). Despite the progress, two major challenges hinder the development of such advanced systems: the wearability and portability of the electromyography (EMG)/ES system for real time unobtrusive use in daily life (*10*–*12*) and the notable variability [skin topography (*12*), muscle anatomy (*13*), and physiological differences (*14*)] among individuals. Wearability directly affects user comfort, patient adherence, and long-term usability, while biological variability influences signal quality, stimulation precision, and ultimately, treatment efficacy (*1*, *4*). For instance, variations in skin structure can affect electrode adhesion and signal quality (*12*, *13*), while differences in muscle mass and distribution can impact the accuracy of EMG acquisition and the efficacy of ES (*15*). Therefore, to fully unlock the potential of closed-loop ES systems, there is a pressing need for personalized, adaptive platforms that can account for user-specific anatomical and physiological differences.

**Fig. 1.**
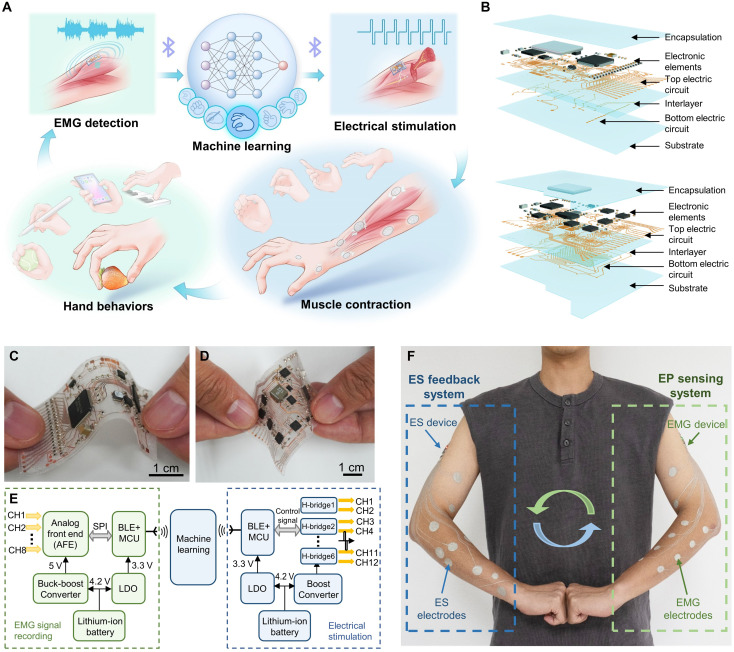
Closed-loop electrical signals sensing-stimulation system. (**A**) Schematic of the closed-loop electrical signals sensing-stimulation system designed to analyze and stimulate hand gestures. (**B**) Exploded-view illustration of the soft circuit modules of the sensing and stimulation device. (**C**) Photograph of the electromyography (EMG) sensing circuit module. (**D**) Photograph of the electrical stimulation (ES) circuit module. (**E**) Circuit diagram of the close-loop control system. (**F**) Schematic illustration of a subject wearing the close-loop sensing and stimulation system.

To address these challenges, we introduce a personalized on-skin network specifically designed for the closed-loop sensing-stimulation (CLSS) system, capable of real-time EMG detection and targeted ES of muscle activities (Fig. 1A). Operating within a wireless communication framework, this system enables seamless kinesthetic feedback between the user and external control systems, supporting applications in motor rehabilitation, interactive training, and remote control. Conventional wet electrodes, such as poly(3,4-ethylenedioxythiophene):polystyrene sulfonate hydrogels, are unsuitable for this application due to dehydration-induced adhesion loss and limited customizability (*16*). At the core of our system is a set of custom-designed, conformal drawn-on-skin (DoS) dry electrodes (*17*–*19*), formulated using high-conductivity silver flakes composites (*20*). These electrodes exhibit excellent skin adhesion, and mechanical compliance, accommodating diverse skin topographies and ensuring stable, high-fidelity EMG acquisition and ES delivery across users. By incorporating machine learning algorithms, we augment the system’s functionality and adaptability (*21*, *22*). The system can accurately differentiate EMG patterns associated with various hand gestures and motor behaviors, enabling automatic recognition and optimized stimulation in real time. Furthermore, anatomically informed modeling of forearm muscle distribution allows us to precisely tailor electrode placement and stimulation parameters, targeting muscle groups at different positions and depths. This capability enhances both selectivity and efficacy of muscle modulation. Our skin-integrated, wireless CLSS platform enables real-time, closed-loop muscle control, offering transformative potential for precision rehabilitation, interactive training, and virtual reality–based motor assistance. Its thin, soft, and wearable form factor ensures excellent user comfort and mobility, making it suitable for applications ranging from home-based therapy and athletic training to immersive digital environments.

## RESULTS

### On-skin CLSS system design

Our CLSS system enables bidirectional EMG sensing and muscle ES activation. The system consists of a pair of skin-integrated input-output devices equipped with customized designed electrodes, which are related to the individual anatomy structure, for EMG measurement and muscle ES (Fig. 1A). Figure 1B shows the schematic diagram of the EMG recording and ES patches, and both the devices are composed of a layer-by-layer structure. The control circuits consist of two electric circuit layers, where vias are soldered through holes in a dielectric polydimethylsiloxane (PDMS) layer (~200 μm thick) to separate the circuit layers (fig. S1). Chips are soldered onto the top layer, followed by encapsulation with an ultrathin PDMS layer (~100 μm thick). Figure 1C shows the EMG recording patch, with its separated structure detailed in fig. S2. The ES patch provides sensation/force assistance to the user’s hand by applying ES to the forearm muscles via customized DoS electrodes (Fig. 1D and fig. S3). Both the EMG and ES patches could be attached on skin (fig. S4) and communicate via Bluetooth (Fig. 1E). The CLSS system architecture comprises several parts: the signal acquisition and processing module, the machine learning module, and the ES module. The signal acquisition and processing module in EMG device captures and processes muscle activity signals, which are then transmitted to the computer for machine learning. The results obtained from machine learning are then transmitted to the ES module, which sends ES to activate the corresponding muscles. Up to eight pairs of DoS electrodes can be connected to the EMG device for signal recording (fig. S5A). The forearm, which houses the muscles responsible for finger movements, can accommodate up to 12 pairs of DoS electrodes, allowing for muscle ES at arbitrary positions (fig. S5B). The ES module triggers muscle contraction. As depicted in Fig. 1F, the EMG and ES devices are attached to the user’s left and right hands, respectively. This setup allows the movements of the right hand to be modified by the behavior of the left hand, which could be particularly useful for training individuals with paralysis.

### Fabrication of DoS electrodes

**Fig. 2.**
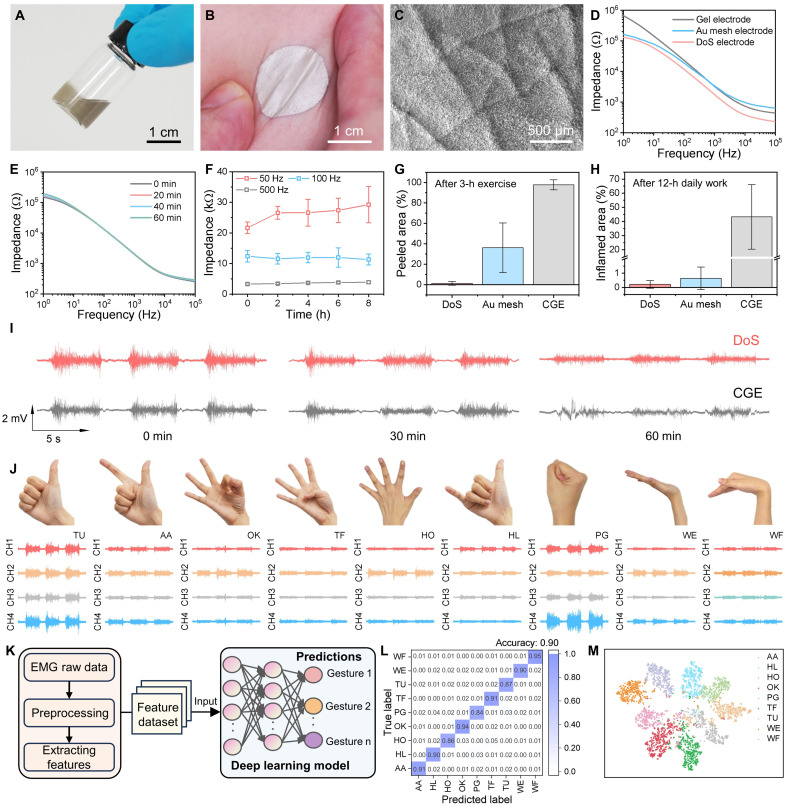
EMG detection system. (**A**) Photograph of drawn-on-skin (DoS) inks. (**B**) Conductive and deformed DoS electrodes on the skin. (**C**) SEM image of microstructure of the skin surface coated with ink. (**D**) Skin impedance of three conductive electrodes: gel electrode, Au mesh electrode, and DoS electrode. (**E**) Skin impedance curves over 1 hour. (**F**) Impedance changes at 50, 100, and 500 Hz, over an 8-hour period. (**G**) Peeling areas of DoS electrode, Au mesh electrode, and commercial gel electrode (CGE) after 3 hours of exercise, *n* = 12. (**H**) Inflamed skin areas coated with each electrode type after 12 hours of daily activity, *n* = 12. (**I**) EMG signals detected by DoS electrodes and gel electrodes over a 1-hour period. (**J**) EMG signals from four channels detected during different hand gestures, including thumb up (TU), acute angle (AA), OK, thumb flexed (TF), hand open (HO), hang loose (HL), power grip (PG), wrist extension (WE), and wrist flexion (WF). (**K**) Diagram of the deep learning model. (**L**) The proposed deep learning model achieved 90% accuracy on test data for nine gestures classes. (**M**) Scatter plot of dimensionally compressed clusters for the classification of nine gesture types.

Because of variations in muscle distribution and the complex morphology of skin among individuals, electrodes with customized geometry would be the solution for precise ES. So, we used DoS silver inks for precise electrode placement. The ink was prepared by mixing liquid band solvent with silver flakes (fig. S6), which exhibit high conductivity and full biocompatibility, making them ideal for painting or drawing electrodes directly onto the skin (Fig. 2A). Using a brush or pen, the inks can be customized deposited onto the skin, conforming to its surface morphology and forming a thin, flexible, and deformable interface that functions as both EMG detection and ES electrodes (Fig. 2B and movie S1). We thoroughly characterized the mechanical, electrical, and writing properties of the DoS ink, confirming its efficacy for skin EMG and ES applications. The sheet resistance of the DoS ink drawn on the skin was measured at 0.89 Ω/sq., demonstrating its excellent conductivity for electrical applications (fig. S7). The scanning electron microscopy (SEM) image of the ink interfacing with a PDMS-based skin replica (Fig. 2C) reveals that the ink effectively fills the crevices of the skin surface (fig. S8), resulting in robust adhesion by mechanically conforming to microscopic surface irregularities. This approach also allows for the fabrication of electrodes in various shapes, sizes, and densities (fig. S9), and the drying time of DoS electrodes is correlated with the amount of ink applied (fig. S10). By adjusting the electrode size, density, and overall arrangement at the point of care, the DoS electrode arrays demonstrate adaptability to different muscles and hand gestures. A crucial factor is the stable attachment of DoS electrodes to the skin even under deformation. When the skin undergoes substantial deformation, the electrodes remain securely attached (figs. S11 and S12 and movie S1).

To validate the performance of the DoS electrodes, we conducted impedance measurements and compared them with other typically used electrodes, including Au-mesh electrodes and commercial gel electrodes (CGEs). Each pair of electrodes was placed on the flexor muscle group of subjects to measure skin-electrode impedance. The results showed that the dry DoS electrodes exhibit superior skin-electrode interfacial properties with low impedance compared to the CGE (Fig. 2D). Impedance measurements were taken at various frequencies every 20 min after electrode application, demonstrating that the impedance remained stable over time (Fig. 2E). In addition, resistance measurements across multiple samples reveal minimal variation, confirming consistent and reliable conductivity (fig. S13). Furthermore, impedance spectra were recorded every 2 hours for up to 8 hours, revealing negligible variation (Fig. 2F). The resistance variation was less than 0.5 Ω during deformation up to 30% strain, with a relative resistance change <20% after 1000 stretching cycles at 30% strain (fig. S14). Moreover, the resistance remained lower than the skin impedance. When users engaged in 3 hours of exercise, the adhesive properties associated with peeling areas were assessed (Fig. 2G). The DoS electrodes adhered well to the skin surface, showing no obvious peeling even in the presence of sweat, whereas the Au-mesh electrodes exhibited weak adhesion, leading to obvious delamination (fig. S15). In a 12-hour daily work test, both the DoS and Au-mesh electrodes demonstrated great air permeability and skin comfortability compared to CGE (Fig. 2H and fig. S16). The ability to easily draw or erase DoS electrodes provides on-demand tunability, allowing comprehensive capture of muscle activity over large areas and enabling customizable ES for personalized care and treatment. In addition, the DoS electrodes maintained better EMG signal quality compared to CGE electrodes after 60 min of exercise, likely due to their strong conformability (Fig. 2I).

Conductive DoS lines could be directly drawn on the skin by a ballpen (fig. S17 and movie S2) or patterned with a stencil, achieving a linewidth resolution as low as 200 μm (fig. S18A). SEM imaging on PDMS substrates confirms the formation of a uniform and stable DoS ink line (fig. S18B). The connecting lines between electrodes can be written on an insulating layer to prevent signal interference during transmission (fig. S19A). The printed lines show good electrical conductivity, where a 6-cm-long and 1-mm-wide conductive line exhibits a resistance of <10 Ω and maintains conductivity even under twisting and stretching (fig. S19, B and C). The skin impedance with an insulating layer approach 10^8^ Ω at frequencies below 100 Hz, which is approximately three orders of magnitude higher than that of electrodes without the insulating layer (fig. S19D). Moreover, this rapid fabrication of DoS circuits enables on-demand reconfiguration, such as establishing electrical connections with light-emitting diodes at different locations (fig. S20). Connections between conductive lines and metal electrodes can be achieved using conductive adhesive tape or standard cables designed for commercial equipment, with resistance <10 Ω (fig. S21). In skin-integrated electronics, the Z-directed tape’s ability to conduct electricity solely through its thickness enables efficient transmission of electrical signals between a series of DoS electrodes and their corresponding metal electrodes.

### EMG signals analysis

Traditional EMG acquisition devices typically rely on wired connections to transmit collected EMG data to instruments for analysis. However, these wired connections can cause obvious motion artifacts, due to the relative movement between the electrode and the skin, degrading the quality of the collected data. In contrast, our CLSS system wirelessly transmits collected EMG data and ES comments via Bluetooth, with all electrodes and connections attached on the skin. EMG signals could be used by feature extraction and pattern recognition for analyzing hand gestures (*23*, *24*).

Recently, deep learning algorithms have gained intensive attention for gesture recognition applications. Traditional methods often focus on single-channel signals, potentially overlooking the rich information present in multichannel EMGs. To leverage the correlation in multichannel EMG signals, we extracted features across multiple channels. We collected EMG signal detection from four pairs of electrodes (*d* = 2 cm), which were placed on the dorsal and volar aspects of the forearm, as depicted in fig. S22. We studied nine hand motions involving flexion and extension of the wrist and five fingers, as illustrated in Fig. 2J. These gestures included thumbs up (TU), acute angle (AA), OK, thumb flexion (TF), hand open (HO), hang loose (HL), power gripping (PG), wrist extension (WE), and wrist flexion (WF). The bursts of muscle activity were clearly distinguishable from the baseline, demonstrating successful capture of summed motor unit activity. We recorded data from 13 healthy adult subjects during data acquisition. After signal acquisition, we applied preprocessing to facilitate feature extraction (Fig. 2K). A 10- to 500-Hz band-pass filter was used to remove baseline wander and high-frequency noise. We used 10-s sliding windows with a Hamming window for data segmentation, followed by normalization. This process runs asynchronously in the background to compute stable statistics (e.g., moving average and SD), enabling adaptive normalization and drift compensation. Eighteen features were extracted from each channel segment, based on the signal’s morphological, time-domain, and frequency-domain characteristics. The 14 time-domain features include mean energy, enhanced average value (*25*), average amplitude change (*26*), maximum fractal length (*27*), skewness, kurtosis, variance, SD, root mean square, zero crossing rate, signal integration, the absolute value of the summation of square roots (*28*), Willison amplitude (*29*), and log-detector (*29*) (text S1). For frequency-domain features, we used four indicators: the energy ratio in three frequency ranges (below 20 Hz, 20 Hz to 50 Hz, and above 50 Hz) and the median frequency of the power spectral density.

The processed data were randomly shuffled and split into 80% for training and 20% for testing. The three-dimensional (3D) dataset served as input to the deep learning model. Convolutional neural networks are powerful tools for feature extraction and fusion. Compared to deep convolutional neural networks, ShuffleNetV2 (*30*) offers advantages such as lower complexity and higher accuracy. Therefore, we implemented an improved model tailored to our data, achieving strong performance. The model architecture includes multiple convolutional layers, MaxPooling layers, Shuffle Block Unit One, Shuffle Block Unit Two, Global Average Pooling layers, and fully connected layers, as shown in fig. S23. The detailed structures of Shuffle Block Unit One and Shuffle Block Unit Two are presented in fig. S24. The training loss gradually converged after 150 epochs, reaching an accuracy of 96.14% on the training set. Because of its superior ability to learn from complex data, the proposed deep learning model achieved 90% accuracy on the testing data for predicting nine gesture classes. The evaluation results for the testing data are illustrated in Fig. 2L as a confusion matrix. To visualize the model’s classification performance, we applied the t-distributed stochastic neighbor embedding algorithm to reduce the data dimensionality to two dimensions. We plotted scatter points from different classes before and after training. As shown in the scatter plot in fig. S25, the different classes of data overlapped without clear boundaries before classification. After model training and prediction, we recorded the output from the first fully connected layer and applied the t-distributed stochastic neighbor embedding method. Figure 2M demonstrates that the data clusters were successfully separated by the model. After processing by the deep learning model, the predicted gestures were sent to the ES device for further action.

### ES strategy

**Fig. 3.**
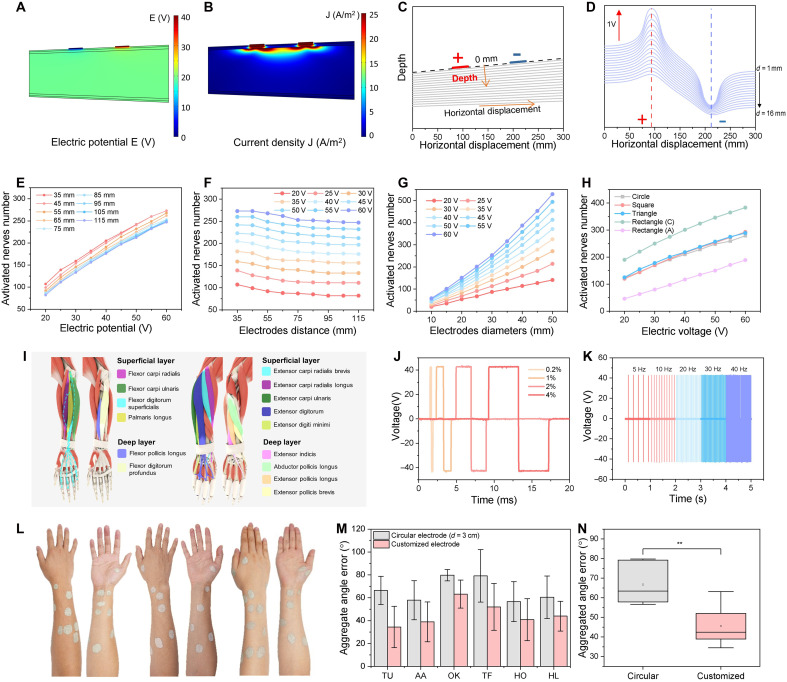
Electrical muscle stimulation. (**A**) Electric potential and (**B**) current density distribution in the electrical simulation model of forearm. (**C**) Schematic diagram of nerve fibers positioned at depths ranging from 1 to 16 mm beneath the skin, and the corresponding (**D**) generated extracellular voltage distributions. (**E**) Simulation results showing a positive correlation between the number of activated nerves and the electric potential delivered by the electrodes. (**F**) Increasing the distance between electrodes leads to a gradual reduction in the number of activated nerves. (**G**) Increasing the electrode diameter substantially enhances the number of activated nerves. (**H**) The neural activation effects of circular, square, and triangular electrodes were comparable across different electrode voltages. (**I**) Schematic diagram of different muscles in the forearm. Electrical pulse with (**J**) different pulse widths and (**K**) different frequencies produced by the ES device. (**L**) Images of personalized electrodes drawn on users’ skin. (**M**) Aggregated angle errors and (**N**) statistic difference of six gestures activated by commercial electrodes and DoS electrodes. Square, mean; center line, median; box limits, upper and lower quartiles; *n* = 5, **P < 0.01.

Precise control over nerve activation is critical for electrical muscle activation. Computer simulations have emerged as essential tools for optimizing ES protocols by systematically analyzing the basic relationships between stimulation parameters, electrode design, and nerve activation patterns. By leveraging simulation models, researchers can design customized stimulation protocols tailored to individual anatomical differences and therapeutic goals, ensuring both precision and efficacy. In this study, we developed a 3D multilayer arm model using finite element methods, integrated with a mammalian neural model, to optimize the placement of customized electrodes (text S2). This model facilitates comparative analysis of various electrode designs and ES parameters, as well as their effects on neural activation (*31*, *32*). The simulation of nerve fiber in the arm by transcutaneous ES involves a two-step process (*33*–*36*). First, the finite element method calculates the potential distribution in the arm using a volume conductor model, allowing evaluation of how ES influences the electric field (Fig. 3A) and current density (Fig. 3B) distribution. Second, nerve fibers located at depths ranging from 1 to 16 mm beneath the skin (Fig. 3C) are used to analyze extracellular voltage distributions along the nerves. These distributions exhibited pronounced curvature beneath the electrodes, indicating strong depolarization that triggers muscle contractions in the corresponding regions (Fig. 3D). Notably, fibers closer to the skin surface experience greater extracellular voltage fluctuations due to the spatial characteristics of the electrical potential distribution.

The activation of nerve fibers is closely tied to the configuration and placement of electrodes. Initially, electrodes with a 30 mm diameter were horizontally aligned and spaced 35 to 115 mm apart, as shown in fig. S26. Data from Fig. 3E and figs. S27 to S28 indicate a predominantly positive correlation between the number of activated nerves and the electric potential delivered by the electrodes. This suggests that the muscle area activated beneath an electrode expands with increasing applied voltage. These findings provide valuable guidance for selecting appropriate voltage parameters to target specific muscles, such as superficial or deeper muscles. Simultaneously, increasing the distance between electrodes results in the gradual reduction in the number of activated nerves, likely due to the diminished electric potential caused by the longer conductive path along the arm (Fig. 3F and fig. S29). This highlights the need to optimize electrode spacing to balance stimulation efficiency and user comfort, as closer spacing enhances activation efficiency while larger spacing reduces activation strength. It is evident that nerve activation is more strongly influenced by the applied voltage than by the distance between electrodes. To further investigate spatial effects, circular electrodes of varying diameters were applied to the arm with a fixed spaced 75 mm apart, as shown in fig. S30A. Data from Fig. 3G indicates that increasing electrode diameter markedly enhances the number of activated nerves, an effect that is further amplified at higher voltages. Figure S30 (B to F) demonstrates that as the electrode coverage area increases, the number of activated nerves beneath it also increases. Unlike increasing the electrode voltage, which deepens the activation area, enlarging the electrode size primarily increases the number of superficial nerves activated around the circumference of the arm, with minimal effects on depth. This finding provides guidance for electrodes design, suggesting that larger electrodes can maximize spatial coverage while minimizing unnecessary stimulation intensity.

The impact of electrode shape on ES was investigated using electrodes configured as circles, squares, triangles, and rectangles, as shown in fig. S31. To ensure a fair comparison, all electrode shapes were adjusted to the same surface area and positioned 75 mm apart. As depicted in Fig. 3H, circular, square, and triangular electrodes produced comparable levels of neural activation across different voltages. However, rectangular electrodes showed obvious variation in nerves activation depending on their orientation. When the long edge of the rectangular electrode was aligned circumferentially, it activated a substantially greater number of nerve fibers (fig. S31, J to L). Conversely, when the long edge was oriented axially, nerves activation was minimal (fig. S31, M to O). This difference is attributed to the axial arrangement of nerves in the arm, where a circumferentially orientation allowed the electrode to overlap with more nerve fibers, thereby enhancing activation (*36*–*38*). In addition, the reduced length of the rectangular electrode in the axial direction slightly contributed to decreased fiber activation (figs. S31, P to R). Notably, the circumferential orientation of the electrode confirmed that the activation area was primarily localized beneath the electrode (fig. S32). Moreover, the polarity of the electrode played an important role in activation spread. Negatively charged electrodes (cathodes) induced a broader area of nerve fiber activation compared to positively charged electrodes (anodes). In ES, the cathode is more effective in inducing neural activation because it attracts positive ions and repels negative ions, reducing the extracellular potential near the nerve membrane. This shift leads to relative depolarization, bringing the membrane potential closer to the threshold for action potential generation, thereby facilitating signal transmission. In contrast, the anode increases extracellular potential, resulting in hyperpolarization of the neuronal membrane, which inhibits depolarization, thereby making neural activation less effective compared to stimulation by the cathode (*39*). These findings highlight the importance of tailoring electrode shapes and orientations to match anatomical structures for optimizing activation efficiency and spatial precision.

### Electrical muscle stimulation

The forearm was selected as the stimulation site because it houses many of the muscles responsible for finger and wrist movements. Figure 3I and fig. S33 illustrate the anatomical distribution of forearm muscles involved in controlling hand gestures (*40*). These muscles enable precise and joint-specific movements. On the superficial volar side, the flexor carpi radialis and flexor carpi ulnaris flex and adduct the wrist, while the palmaris longus flexes the wrist and tenses the palmar aponeurosis. The flexor digitorum superficialis flexes the proximal interphalangeal and metacarpophalangeal (MCP) joints of the index, middle, ring, and little fingers. In the deep volar layer, the flexor digitorum profundus flexes the distal interphalangeal and MCP joints of the same fingers, and the flexor pollicis longus flexes the interphalangeal and MCP joints of the thumb. On the dorsal side, the extensor carpi radialis longus and brevis extend and abduct the wrist, while the extensor carpi ulnaris extends and adducts it. The extensor digitorum extends the fingers and wrist, and the extensor digiti minimi specifically extends the little finger. In the deep dorsal layer, the extensor indicis extends the index finger; the abductor pollicis longus abducts the thumb at the carpometacarpal (CMC) joint and acts as an accessory extensor. The extensor pollicis longus extends the interphalangeal, MCP, and CMC joints of the thumb, while the extensor pollicis brevis extends the MCP and CMC joints. As shown in the cross-sectional view of muscle distribution (fig. S34), the muscles in the forearm are not strictly aligned along the arm’s longitudinal axis. Instead, the forearm features a complex arrangement of superficial and deep muscles, each playing a critical role in enabling precise and coordinated hand movements. Simulation results reveal that superficial muscles are more easily activated by ES, while deeper muscles, although activatable, require higher voltages or strategic electrode placement for effective stimulation, which often stimulate untargeted nearby superficial muscles. In addition, the anatomical distribution of muscles, as illustrated in Fig. 3I, underscores the importance of electrode orientation, particularly for muscles that do not follow the arm’s longitudinal axis. Aligning electrodes with the direction of muscle fibers is crucial for selective and efficient stimulation. By combining anatomical insights with simulation data and tailoring stimulation protocols to accommodate the forearm’s structure complexity, the ES system achieves precise and reliable control of specific gestures by transcutaneous ES, even within this intricate anatomical environment.

ES is generated by an electronic pulse generator and delivered through channels connected to on-skin electrodes. A symmetrical biphasic waveform was chosen for muscle stimulation, as it is widely regarded as the most comfortable and effective method for activating large muscle groups (Fig. 3J). This waveform also minimizes the risk of skin irritation due to its balanced charge delivery. The current required to induce muscle contraction varies among individuals, and the stimulation frequency can be adjusted on the basis of the desired muscular response (Fig. 3K). Although larger electrode pads can improve user comfort and increase tolerance to ES, they often reduce selectivity by unintentionally activating adjacent muscles or failing to deliver sufficient current density for effective stimulation. This lack of selectivity remains a major limitation in muscle ES. Conventional high-density electrode arrays also struggle to achieve the spatial precision required to target individual muscles with complex anatomical geometries. Furthermore, these arrays are typically not reconfigurable in situ, limiting the ability to optimize electrode number and placement during use. Externally triggered hand gestures can be elicited when motor units respond to ES (*4*, *10*, *11*, *36*). Achieving isolated single-finger movements via stimulation of individual motor points is particularly challenging due to the natural coupling of finger motions, and because adjacent joints may passively follow the movement of the stimulated finger. Large electrodes tend to activate broader populations of motoneurons, increasing the risk of coactivating nonsynergistic muscles. While this reduces selectivity, it also lowers the risk of high current densities and decreases the number of independent stimulation channels needed. In contrast, small electrodes produce more localized electric fields, reducing current spread and improving selectivity. However, this comes at the cost of increased system complexity and a higher risk of localized high current density. Thus, precise control of charge distribution is essential for the selective activation of specific motor units.

To validate the effectiveness of our system, we conducted experiments to identify which hand gestures could be reliably controlled using our setup. Participants were stimulated to perform nine predefined hand gestures (Fig. 2J), including TU, AA, OK, TF, HO, HL, PG, WE, and WF (text S3). Intersubject variability, such as differences in forearm circumference, electrode array positioning, and individual physiological responses to ES, was evident across trials. To minimize discrepancies between ES-induced gestures and spontaneous gestures, we optimized electrode selection and positioning for each participant. Optimal electrode configurations varied among individuals due to physiological differences (Fig. 3L). In general, electrodes on the dorsal forearm were used for finger extension, while those on the volar side were used for flexion. Electrode size and shape were customized to each subject to ensure effective stimulation. To compare the performance of customized DoS electrodes with CGEs, we defined an aggregated error metric as the root of the sum of squared differences between joint angles produced by ES and those observed during spontaneous gestures (figs. S35 to S43). This aggregated error was calculated for six gestures using both circular commercial electrodes and individually tailored DoS electrodes (Fig. 3M). Figure 3N summarizes the aggregated errors for the optimal electrode configurations across five participants. Results showed that the customized DoS electrodes achieved substantially lower aggregated angle errors compared to commercial fixed-size electrodes, due to their tailored fit and precise positioning. These findings support the potential of our system for implementing user-specific interaction interfaces, enabling accurate and flexible control of hand motion and gesture performance.

### Applications of muscle control

**Fig. 4.**
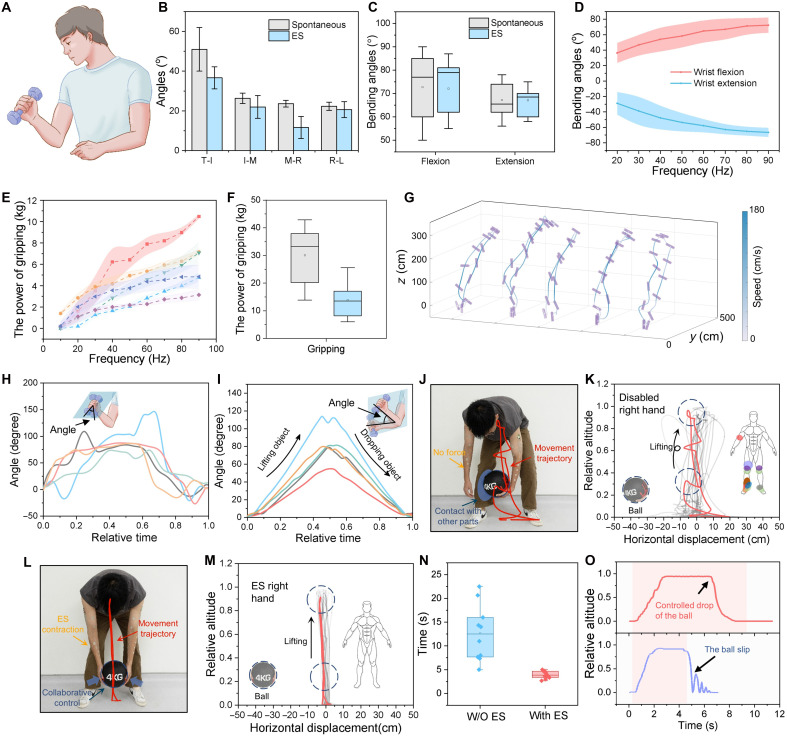
Close-loop control application. (**A**) Schematic image of a participant holding an object. (**B**) Angles between adjacent fingers during hand opening, including the angle between the thumb and the index finger (T-I), the index finger and the middle finger (I-M), the middle finger and the ring finger (M-R), and the ring finger and the little finger (R-L), *n* = 10. (**C**) Maximum bending angles during spontaneous and stimulated WF and extension, *n* = 10. (**D**) Wrist bending angles when activated by ES of different frequencies. (**E**) Gripping force when hand was activated by ES of different frequencies. (**F**) Maximum gripping force during spontaneous and electrically stimulated griping, *n* = 10. (**G**) Object movement trajectories when grasped by a hand activated through ES. (**H**) Angles of objects and (**I**) forearms during the holding process. (**J**) Images of a boy lifting a 4-kg ball using only his left hand and the corresponding (**K**) movement trajectories of the ball. (**L**) Images of a boy holding a 4-kg ball with the assistance of ES on the right hand and the corresponding (**M**) movement trajectories of the ball. (**N**) Lifting time with and without the assistance of the right hand for different individuals, *n* = 10. (**O**) The height trajectory of the ball during the holding process shows that in the upper curve, the ES remains active, maintaining the ball’s position, while in the lower curve, the sudden deactivation of the ES causes the ball to abruptly fall.

The CLSS system presented in this study holds enormous potential for practical applications, such as providing kinesthetic feedback in virtual reality and assisting individuals with hand paralysis in regaining partial motor function. In this context, ES enables users to grasp objects based on detected motor intentions or guided behaviors, offering a promising pathway for rehabilitating individuals who have lost voluntary hand control (Fig. 4A). One key metric for evaluating hand functionality is the angle between adjacent fingers during hand opening, as shown in fig. S39. When comparing ES-induced hand-opening gestures with spontaneous ones, we observed similar angular distributions between fingers, as well as comparable WF and extension angles across participants. However, because of differences in the temporal dynamics of muscle fiber activation between spontaneous and ES-induced movements, the maximum finger opening angles achieved with ES were slightly lower than those achieved spontaneously (Fig. 4B). A similar trend was observed during hand flexion (Fig. 4C), where reduced bending angles were noted under ES. As shown in Fig. 4D, increasing the frequency of ES resulted in larger finger bending angles. This frequency-dependent relationship also extended to grip strength: higher stimulation frequencies produced greater gripping power (Fig. 4E), although the maximum grip force achievable with ES remained lower than that generated through voluntary effort (Fig. 4F).

To further demonstrate real-world applicability, we integrated biceps brachii stimulation with the previously described hand stimulation system to enable users without spontaneous arm movement to grasp and lift objects. Movement trajectories of the lifted cylindrical objects were recorded and analyzed (Fig. 4G, movie S3, and text S4). All participants successfully grasped and lifted objects multiple times, despite lacking voluntary muscle contraction. In Fig. 4G, the short purple lines represent the spatial orientation of the object at different time points, while the continuous line traces the trajectory of the object’s center during the lifting process. The color gradient along the trajectory indicates the movement speed, with darker colors representing faster speeds and lighter colors indicating slower movement. During the lifting process, camera-based tracking was used to extract wrist (Fig. 4H) and elbow (Fig. 4I) joint angles. The results showed clear wrist rotation and rapid elbow flexion, corresponding to ES-induced muscle contractions.

To highlight the importance of bilateral coordination, an additional user study was conducted in which participants were tasked with lifting a 4-kg ball while their right hand was rendered nonfunctional (Fig. 4J, movie S4, and text S5). Without stimulation, participants had to compensate for using other body parts, resulting in irregular and deviated lifting trajectories (Fig. 4K). However, when the right hand was stimulated in response to EMG signals from the left hand, participants were able to lift the ball along a straight trajectory using only their upper limbs (Fig. 4, L and M). The lifting duration was substantially reduced in the ES-assisted condition, emphasizing the value of coordinated bimanual behavior (Fig. 4N). Furthermore, participants were able to maintain their grip on the heavy ball while ES was active, but dropped the ball immediately upon deactivation of stimulation (Fig. 4O). These findings collectively underscore the potential of the CLSS system for assistive and rehabilitative applications, particularly for individuals with hemiplegia or partial motor impairment.

## DISCUSSION

In this study, we developed a wireless skin-integrated personalized CLSS system for detecting EMG signals and delivering ES, tailored to each user’s muscle anatomy with customized electrode placement that securely adheres to the skin. The system comprises two key components: wireless EMG signal detection and ES, both powered by small batteries and connected via Bluetooth communication. Machine learning algorithms are used to classify these signals and accurately recognize various hand gestures from arrayed EMG signals. The optimal electrode positions are determined by minimizing the differences between the joint movements of fingers (across 14 joints) during stimulated and spontaneous hand gestures. Our results demonstrate that the system effectively facilitates multiple hand gestures by applying targeted ES to different areas of the upper extremities. It is important to note that the optimal electrode position and shape are influenced by the geometry of the forearm, as the relative motion of forearm tissues affects the placement of the DoS electrodes. Furthermore, the optimal electrode configurations differ between the palmar and lateral sides of the forearm due to individual variations in the sensory-motor system’s physical layout. The wireless CLSS system holds great potential in physical therapy, rehabilitation, and sports training among others.

## MATERIALS AND METHODS

### Materials and DoS ink preparation

All chemicals were used as received without treatment. DoS ink consists of Ag flakes (10 μm, 99.9%, Sigma-Aldrich) and liquid band solvent (New-Skin, Advantice Health LLC) with the weight ratio of 1:2, stirring for over 30 min. Adhesive layer was brush on the skin using acrylic adhesive glue (Pros-Aide), which is composed of water, acrylic emulsion, glycerol, guar gum, sorbitol, and benzyl alcohol. Liquid bandage is composed of nitrocellulose, benzethonium chloride, amyl acetate, camphor, ethyl acetate, ethyl alcohol, and *n*-butyl acetate.

### Fabrication of DoS electronic tattoo

Conductive electrodes were first drawn directly onto the skin using a brush or ballpoint pen filled with DoS ink and allowed to dry at room temperature or with hot air. Once dried, a liquid bandage was applied with a brush from the electrode sites to the target device regions, forming a conformal insulating layer. A stencil was then used to pattern conductive DoS ink onto the insulating layer, precisely aligned with the copper contact pads of the devices to facilitate electrical connection. After drying and stencil removal, additional conductive traces were hand-drawn with the DoS ink pen to complete the circuit. The time required to draw conductive traces depends on their length and the complexity of the body part. Last, commercial double-sided conductive adhesive tape was used to establish robust electrical connections between the electrodes and conductive traces.

### Device fabrication

The flexible circuit of EMG monitoring and ES devices began with laminating the laser-structured PET sheet to the glass wafer as the mold, followed by pouring PDMS into the mold and curing at 80°C for 10 min. Spin casting PDMS (500 rpm, 30 s) on a temporary glass sheet was followed by curing at 100°C for 10 min. Cu foil (18 μm) was laminated on the prepared PDMS/glass substrate and then structured into the designed pattern by laser cutting (LPKF ProtoLaser U4). The pattern was picked up by water-soluble tape (WST). The first layer of patterned Cu interconnection was directly laminated on the molded PDMS substrate after activating by ultraviolet ozone for 5 min and heated at 80°C for 10 min. The WST was removed by deionized water. Laser-patterned PET stamps (0.2 to 0.6 mm diameter and 1 mm thickness) were placed using thermally sensitive adhesive on the Cu pads. Repeated pouring of PDMS into the mold and curing at 45°C for 12 hours was followed by removing the PET stamps at 60°C, forming the vertical interconnect access through the PDMS (about 200 μm thick). The second layer of the Cu pattern was transferred onto the PDMS in the same way. Soldering the electrical components [microcontroller, resistors, capacitors, Bluetooth low energy (BLE), light-emitting diode, and so on], battery and Cu interconnected pads were carried out using low-melting-point solder paste. The circuit was sealed by brushing a thin layer of PDMS (about 100 μm thick) on it and cured at 80°C for 10 min, followed by removal from the mold. The adhesion between the devices and the skin was achieved using a double-sided medical-grade tape (2477P Medical Tape, 3M) to ensure stable attachment (fig. S4). A peel-off test (Instron 5942, Norwood, MA, USA) was conducted on ex vivo porcine skin tissue, resulting in a peel-off strength of 45 ± 8 N/m.

### Characterization

The micromorphology for the DoS electrodes was observed by an SEM (FEI Quanta 250, USA). The impedance of the interface between skin and different electrodes was determined using a Chemical Impedance Analyzer (Hioki, IM3590). The mechanical properties were evaluated by uniaxial tensile and compressive tests using a universal testing machine (Instron model 5942, Norwood, MA, USA).

### Circuit designs

The entire CLSS system comprises two primary components: the EMG sensing module and the ES module (figs. S44 and S45). These modules communicate wirelessly with a host device via BLE. The EMG sensing module utilizes an Analog Front End (AFE, ADS1299, TI), capable of simultaneously acquiring eight 24-bit EMG signals. A microcontroller unit (MCU; CC2640R2F, TI) processes these signals, packages them, and transmits them in real-time to the host device over BLE. A low-dropout regulator (LDO; TPS76933, TI) converts the 3.7 V battery voltage to 3.3 V to power the MCU, while a buck-boost converter (TPS63002, TI) converts the same 3.7 V battery input to 5 V to supply power to the AFE. A simple filtering network, consisting of capacitors and resistors, is used to attenuate high-frequency noise, thereby enhancing the signal-to-noise ratio.

The ES module also uses the CC2640R2F as both the MCU and Bluetooth communication chip. The TPS76933 is used as the LDO. A boost converter (LT8364, Analog Devices) elevates the 3.7 V battery voltage to 48 V, providing the required voltage for ES. To deliver bipolar pulses for muscle ES, H-bridges (DRV8436, TI) are used to generate modulated signals. Upon receiving instructions from the host device, the MCU generates distinct pulse width modulation waveforms corresponding to different motion patterns on the appropriate channels, enabling targeted muscle ES to produce predetermined movements.

The EMG signals acquired by the wearable device are transmitted to the PC via Bluetooth. Upon receiving the data, the PC processes it and inputs it into a pretrained deep learning model. For data communication, we implemented a WebSocket connection between the server on the PC and the client on the mobile device. The server transmits the prediction results and the down-sampled signals to the mobile application as soon as they are generated and sends the command via Bluetooth back to the device for the control of the ES. Consequently, the results and data can be visualized in real time on the mobile application.

### Ethics statement

All user studies were conducted with healthy adult participants in accordance with protocols (HU-STA-00001654) approved by the City University of Hong Kong. Participants were recruited from the City University of Hong Kong campus via posted notices and email announcements. Before the experiment, each participant received an information sheet describing the study objectives, procedures, potential risks and benefits, and data handling practices, and was given the opportunity to ask questions. Written informed consent was obtained from all participants before any data were collected.

## Supplementary Materials

### The PDF file includes:

Supplementary Text Table S1 Figs. S1 to S45 Legends for movies S1 to S4 References
