# Supplementary material for: Drawn-on-skin electronic tattoo as a closed-loop sensing-stimulation system for the muscles
Source: Sci Adv. 2026 Apr 15;12(16):eaed7673. doi: 10.1126/sciadv.aed7673 (PMC13082325; doi:10.1126/sciadv.aed7673)
Supplement: Supplementary file 1 — Supplementary Text Table S1 Figs. S1 to S45 Legends for movies S1 to S4 References [file sciadv.aed7673_sm.pdf]

Science Advances
AAAS

Supplementary Materials for

Drawn-on-skin electronic tattoo as a closed-loop sensing-stimulation system for the muscles

Ya Huang *et al.*

Corresponding author: Ya Huang, yahuang@fudan.edu.cn; Dong Sun, medsun@cityu.edu.hk; Cunjiang Yu, cunjiang@illinois.edu; Xinge Yu, xingeyu@cityu.edu.hk

Sci. Adv. **12**, eaed7673 (2026)
DOI: 10.1126/sciadv.aed7673

The PDF file includes:

Supplementary Text
Table S1
Figs. S1 to S45
Legends for movies S1 to S4
References

Other Supplementary Material for this manuscript includes the following:

Movies S1 to S4

Supplementary Text

Features extraction of EMG signals

We have extracted multiple features from the acquired EMG signal for gesture recognition. The definitions of various features are expressed as follows.

Mean energy:

$$ME = \frac{1}{N}\sum_{n}|x(n)^2| \quad \text{(S1)}$$

Enhanced average value:

$$EAV = \frac{1}{N}\sum_{n}|(\boldsymbol{x_i})^p| \quad \text{(S2)}$$

$$\begin{cases} p = 0.75, if\ i > 0.2N\ and\ i < 0.8N \\ p = 0.5, otherwise \end{cases}$$

Average amplitude change:

$$AAC = \frac{1}{N}\sum_{n}|x_{i+1} - x_i| \quad \text{(S3)}$$

Maximum fractal length:

$$\mathrm{MFL} = \log_{10}\sqrt{\sum_{n}(x_{i+1} - x_i)^2} \quad \text{(S4)}$$

Standard deviation:

$$SD = \frac{\sum|x - \bar{x}|^2}{n} \quad \text{(S5)}$$

Where $\bar{x}$ is mean value of the data.

Skewness:

$$SKW = \frac{3\bar{x} - M_d}{SD} \quad \text{(S6)}$$

Where M_d is the median value of the data.

Kurtosis:

$$K = \frac{\sum_{i=1}^{n}(x_i - \bar{x})^4}{(\boldsymbol{n} - \boldsymbol{1}) * SD^4} \quad \text{(S7)}$$

Variance:

$$\sigma^2 = \frac{\sum_n(\boldsymbol{x_i} - \boldsymbol{\bar{x}})}{n} \quad \text{(S8)}$$

Root mean square:

$$RMS = \sqrt{\frac{1}{n}\sum_{n}x_i^2} \quad \text{(S9)}$$

Zero crossing rate:

$$ZCR = \frac{1}{2N}\sum_{i=1}^{N}\left|sign\big(x(i)\big) - sign\big(x(i-1)\big)\right| \quad \text{(S10)}$$

Where the sign function is defined as:

$$sign(x(i)) = \begin{cases} 1, & x(i) \geq 0 \\ -1, & x(i) < 0 \end{cases} \tag{S11}$$

Signal integration：

$$SI = \int_0^t x(t)dt \tag{S12}$$

Absolute value of the Summation of Square root:

$$ASS = |\sum_{i=1}^{N} (\boldsymbol{x_i})^{1/2}| \tag{S13}$$

Willison amplitude:

$$WA = \sum_n f\,(x_i - x_{i+1}) \tag{S14}$$

$$f(x) = 1, \quad if\ x \geq threshold$$

$$f(x) = 0, \quad otherwise$$

Log-detector:

$$LD = e^{\frac{1}{N}\sum_{i=1}^{N} \log(|\boldsymbol{x_i}|)} \tag{S15}$$

Energy ratio:

$$ER = \frac{\sum P_{selected}}{\sum P_{total}} \tag{S16}$$

Where $P_{selected}$ is the power spectral density of selected frequency, P_{total} is the power spectral density of signal.

Median frequency of the power spectral density:

$$MFP = \frac{1}{2}\sum_{i=1}^{n} P_i \tag{S17}$$

Where P_i is the power spectral density.

Modeling and simulation of electrical muscle stimulation

We have developed a sophisticated 3D multilayer arm model utilizing finite element methods, integrated with a mammalian neural model, to optimize the positioning of customized electrodes. This model facilitates comparative analysis of various transcutaneous electrical stimulation (ES) parameters and their impact on neural activation. The simulation of nerve fibers in the arm by ES is executed through a two-step process (*33,34,36*). Initially, the finite element method is employed to calculate the potential distribution within the human arm using a volume conductor model. Electrical stimulation is controlled by applied voltage, which generates the resulting electric field distribution and current density in the forearm. Subsequently, the resulting extracellular potentials induced by stimulation are used to predict the action potentials in nerve or muscle fibers.

The arm model (volume conductor) and the resulting scalar potential (V_e) within the tissue and at the electrode interface are governed by the following equations, all simulations were carried out using COMSOL Multiphysics:

$$\nabla \cdot [\sigma \nabla V_e] - \nabla \cdot \left[\varepsilon \nabla \frac{\partial V_e}{\partial t}\right] = 0 \tag{S18}$$

In the model, two stimulating electrodes were represented as highly conductive substrates, with variations in size (diameters ranging from 10 to 50 mm), center-to-center spacing (15 to 115 mm), shape (round, square, triangle, and rectangle), and angular orientation (0° to 180°). These parameters were meticulously chosen during the simulation setup to evaluate their influence on ES performance. The applied electric potential on the electrodes varied between 20 and 60 V. The forearm was modeled as a multi-layered frustum, with a lower base radius of 50 mm and an upper base radius equal to 80% of the lower base. The geometry includes distinct tissue layers—skin (1.5 mm), fat (2.5 mm), and muscle (46 mm)—and extends over a total length of 300 mm (figs. S26A and S26B). Notably, the arm model excludes the skeleton to allow for clearer analysis of the depth of ES penetration and its effects on muscle activation across different regions.

The electrical properties, specifically resistance and relative permittivity, assigned to each tissue type and to the electrodes, are detailed in Table S1 (*41*, *42*). To accurately reflect the anisotropic characteristics of muscle tissue, different values were used for axial and radial directions; the radial resistivity was set to three times the axial resistivity ($3 \times \rho_{axial} = \rho_{radial}$) (*43*). The time-dependent electrical potential fields derived from the finite element model were subsequently interpolated along lines at varying depths to simulate the positions of nerve bundles, which were uniformly distributed across the cross-section (x-y plane) of the arm model. For example, when circular electrodes with a diameter of 30 mm were placed 115 mm apart, the resulting electric potential and current density distributions are shown in figs. S26C and S26D.

Chamber models are essential for understanding how applied electric or magnetic fields influence specific target neurons. A foundational example is McNeal's compartmental model for myelinated nerve fibers, which focused on their subthreshold responses to external point-source stimulation (*32*). In his approach, McNeal represented myelinated nerves using the equivalent circuit of a Ranvier node, assuming the myelin sheath acted as a perfect insulator. This formed the basis of the widely used electrical network model for myelinated nerves. In contrast, our model treats the myelin sheath as a passive circuit element rather than a perfect insulator, allowing for a more realistic representation of its electrical behavior. The membrane voltage in each compartment is defined as:

$$V_n = V_{i,n} - V_{e,n} - V_{rest} \tag{S19}$$

where V_n is the transmembrane potential, $V_{i,n}$ and $V_{e,n}$ are the intracellular and extracellular potentials, respectively, and V_{rest} is the resting membrane potential (*31*). This formulation leads

to a set of differential equations that govern the time-dependent dynamics of V_n across each compartment.

$$c_m \frac{\partial V_n}{\partial t} = -I_{ion,n} + \frac{V_{n-1} - 2V_n + V_{n+1}}{R} + \frac{V_{e,n-1} - 2V_{e,n} + V_{e,n+1}}{R} \tag{S20}$$

To determine the membrane voltage, it is necessary to calculate the ionic current $I_{ion,n}$ for each compartment. In our model, the internodal regions are treated as passive membranes, characterized by a constant membrane conductance ($G_{m,n}$). For the nodes of Ranvier, we utilized the CRRSS model (Chiu-Ritchie-Rogart-Stagg-Swenney) (*44, 45*), which captures the nonlinear gating dynamics of ion channels in the membranes of unmyelinated neurons. This model incorporates both sodium and leakage currents, while potassium channels are deliberately excluded, based on experimental evidence indicating that potassium currents play a limited role in the excitation of myelinated mammalian nerves. The instantaneous changes in membrane voltage at each node and internode, in response to an externally applied field, can be effectively estimated using an activation function, denoted as f (*31, 46*). This formulation enables a dynamic and physiologically relevant assessment of neuronal response to ES, providing critical insights into neural behavior under varying stimulation conditions.

$$f_n = \frac{1}{C_{m,n}} \frac{V_{e,n-1} - 2V_{e,n} + V_{e,n+1}}{R_a} \tag{S21}$$

The activation function f utilizes the extracellular voltage V_e along the length of the neuron as input to identify the location of the strongest depolarization at the onset of stimulation without requiring detailed modeling of ion channel kinetics. Additionally, f provides an approximate estimation of the excitation threshold for the targeted nerve fiber. When a single stimulus pulse is applied to a nerve fiber initially at rest, the activation function exhibits a peak at the location where depolarization is most likely to initiate an action potential. This approach offers a computationally efficient method for predicting neural activation sites. The nerve fiber model, including the implementation of the activation function, has been successfully developed using MATLAB 2023 (The MathWorks Inc., Natick, MA).

Table S1.

The parameters used in TES simulation.

Tissues	Resistivities (Ωm)	Relative permittivity	Thicknesses (mm)
Electrode interface	300	1	0.5
Skin	700	6000	1.5
Fat	33	2500	2.5
Muscle (axial)	3	120000	46
Muscle (radial)	9	40000	46

Analysis of different hand gestures

Due to the anatomical structure of the arm, as illustrated in Fig. 3I, fig. S33, and fig. S34, accurately activating the appropriate muscles is crucial for eliciting specific hand gestures through ES.

Thumbs up is demonstrated in fig. S35. This gesture is primarily controlled by the abductor pollicis longus (APL), extensor pollicis longus (EPL), and extensor pollicis brevis (EPB) muscles. Anatomical cross-sectional views show that these muscles are located near the superficial layer of the forearm, especially close to the wrist. Therefore, electrodes for thumb extension should be positioned near the wrist to effectively stimulate these muscles. Flexion of the other four fingers is governed by the flexor digitorum superficialis (FDS) and flexor digitorum profundus (FDP), which can be stimulated at the mid-forearm. However, due to the proximity of the flexor carpi ulnaris (FCU), which lies more superficially than the FDS and FDP, its activation is often unavoidable during stimulation, leading to involuntary wrist flexion.

Acute angle gesture, shown in fig. S36, requires extension of both the thumb and index finger. This involves stimulation of the extensor indicis (EI), along with APL, EPL, and EPB. These muscles are also located near the wrist, making them accessible via electrodes placed in that region. However, the EI is anatomically adjacent to the extensor digitorum (ED) and extensor digiti minimi (EDM), which may also be unintentionally activated. Fortunately, since the distal portions of ED and EDM are at the muscle ends near the wrist, their contribution to finger extension is limited. Additionally, the flexion of the index finger by FDS and FDP must be carefully modulated, as these muscles also control the flexion of the other fingers.

OK gesture, illustrated in fig. S37, involves flexion of the thumb and index finger, controlled by the flexor pollicis longus (FPL), FDS, and FDP. Since FPL exclusively controls thumb flexion, precise stimulation can reliably produce this movement. However, FDS and FDP also control the flexion of the middle, ring, and little fingers, which is undesirable in this gesture. To counteract this, stronger stimulation of ED and EDM is applied to extend the middle, ring, and little fingers.

Thumb flexion, shown in fig. S38, requires only the activation of FPL, which is not functionally coupled with the other fingers. Therefore, extension of the remaining four fingers can be independently achieved by stimulating EI, ED, and EDM. Given that EI lies deeper within the arm, ED and EDM are preferable targets for efficient finger extension.

Hand open, depicted in fig. S39, involves simultaneous extension of all fingers. All associated muscles, including ED, EDM, EI, APL, EPL, and EPB, are located on the dorsal side of the forearm and are relatively superficial. As such, hand opening can be reliably achieved using a pair of electrodes placed dorsally, provided the stimulation exceeds the activation threshold.

Hang loose gesture, shown in fig. S40, is more challenging to elicit via ES. This gesture requires flexion of the index, middle, and ring fingers (controlled by FDS and FDP), while the little finger must remain extended. However, activation of FDS and FDP often leads to unintended flexion of the little finger. To counterbalance this, a stronger stimulus must be applied to EDM in order to ensure extension of the little finger. Meanwhile, thumb extension can be achieved by stimulating APL, EPL, and EPB.

Power gripping, illustrated in fig. S41, is relatively easy to induce by simultaneously stimulating FDS, FPL, and FDP on the volar side of the forearm, since all fingers are required to flex. However, the grip strength generated through ES is typically weaker than natural gripping. In addition, unintentional activation of the FCU may lead to unwanted wrist flexion.

Wrist extension (WE), shown in fig. S42, can be achieved by concurrently stimulating the extensor carpi radialis brevis (ECRB), extensor carpi radialis longus (ECRL), extensor carpi

ulnaris (ECU), and ED on the dorsal forearm. This movement may also induce simultaneous finger extension, contributing to a hand-open gesture.

Wrist flexion (WF), shown in fig. S43, is produced by stimulating the flexor carpi radialis (FCR), FCU, and palmaris longus (PL) muscles on the volar side of the forearm.

User study of raising the object

To demonstrate our system's capability to control hand gestures through ES, we conducted a user study in which the right hand of one participant (the "follower") was actuated to lift an object by replicating the hand movements of another participant (the "initiator"), as shown in Supplementary Movie S3. An EMG recording device and an ES device were independently attached to the initiator and the follower, respectively. A total of 10 participants took part in the study (4 females, aging 24-35 years old), each performing the task of lifting a cylindrical object for more than 5 times. This task involved sequential activation of muscles for grasping, elbow flexion, elbow extension, and hand opening, all triggered by ES based on the initiator's movements.

Participants were instructed to relax their right arm and hand, which were placed flat on a table. A plastic cylinder with a diameter of 3 mm and a length of 11 mm was positioned in each participant's hand prior to stimulation. During the experiment, the EMG device detected the grasping motion of the initiator in real time. This signal was then used to activate the follower's corresponding muscles via ES. As a result, the follower executed a series of coordinated movements: grasping the cylinder, lifting it by flexing the elbow, lowering it by extending the elbow, and finally releasing it by opening the hand.

The movement trajectory of the cylinder during the followers' grasping actions was recorded using two orthogonally positioned smartphones with a resolution of 1920 × 1080 pixels, capturing both front and side views. This setup enabled the extraction of spatial and angular information of the cylinder through image processing techniques. An x-y-z coordinate system was established, defined by the plane of the participant's elbow movement and the tabletop. The cameras were aligned along the x- and y-axes and calibrated prior to the experiment to determine the pixel-to-distance conversion ratio. All image data were processed using MATLAB 2023 (The MathWorks Inc., Natick, MA), allowing for the calculation of the cylinder's 3D position, its orientation, and the angle between the participant's forearm and the tabletop.

User study of holding the ball

To evaluate the system's potential for facilitating coordinated bilateral hand movements, we conducted a user study in which participants were assisted in lifting a 4 kg ball under the condition that their right hand was non-functional (Fig. 4J). A total of 10 participants took part in this experiment. Each participant wore an ES patch on their right forearm, configured to activate the muscles responsible for grasping, and an EMG detection patch on the left forearm to monitor voluntary muscle activity. The ball was placed on the ground in front of the participants. The entire experiment was recorded using a front-facing smartphone camera, as shown in Supplementary Movie S4. The position and movement trajectory of the ball were later extracted from the video using MATLAB.

In the first phase of the experiment, participants were instructed to lift the medicine ball without using their right arm, relying solely on their left arm and, if necessary, other parts of the body. Due to the weight and size of the ball, it was difficult to lift it with one hand alone. As a result, participants often compensated by using additional body movements, leading to varied and inconsistent lifting trajectories (Fig. 4K).

In the second phase, the EMG patch on the left arm was used to detect voluntary muscle contractions associated with hand grasping. Upon detection of these signals, commands were relayed to the ES device on the right arm, which then stimulated the corresponding muscles to produce hand grasping (Fig. 4L). Participants were instructed to place both hands on either side of the ball and actively flex their left wrist during the grasping motion. Once the EMG signal was detected, the system triggered ES on the right arm, enabling it to mirror the grasping action and generate force on the opposite side of the ball.

With both hands effectively coordinated through this closed-loop sensing-stimulation system, participants were able to lift the medicine ball without relying on compensatory movements from other body parts. In contrast to the first phase, the movement trajectory of the ball during this coordinated lifting was more linear, and the lifting process was smoother and more stable (Fig. 4K).

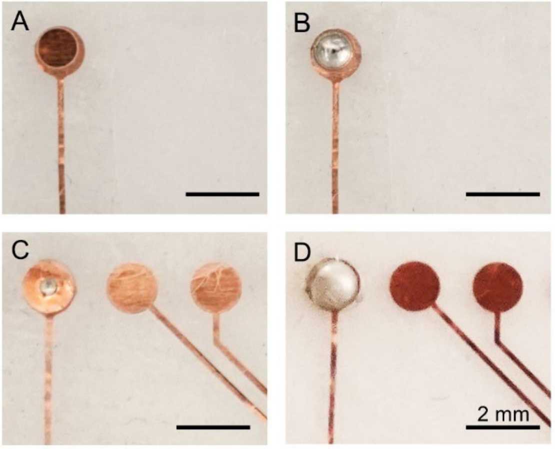

Fig. S1.

Photograph of the through-hole fabrication process. (**A**) Laser-cut Cu/PI bottom conductive lines attached to the PDMS substrate, with an insulating PDMS layer placed over the bottom conductive lines, featuring pre-cut holes. (**B**) Tin solder applied to the electrode that should be connected with top layer. (**C**) Top electronic circuit was attached on the separated bottom electronic circuit. (**D**) Top circuit was connected with bottom circuits by soldered through the small hole in the electrode.

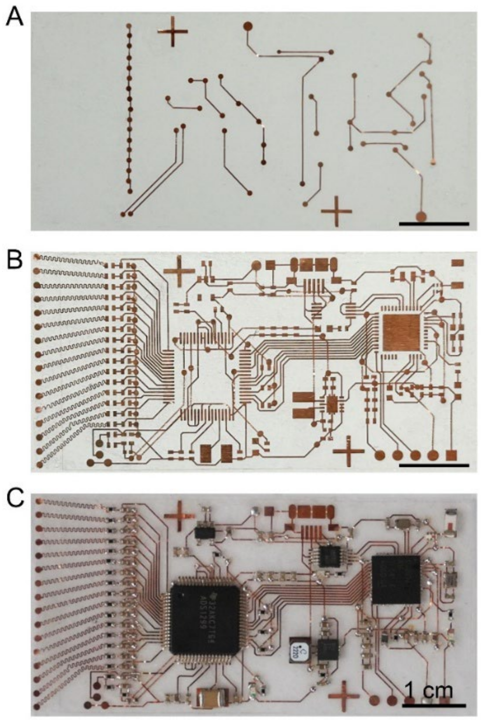

Fig. S2.

Photograph of the EMG detection device fabrication process. (**A**) Bottom electric circuits. (**B**) Assembled top and bottom electric circuits before soldering. (**C**) Soldering of all through-holes and electric components.

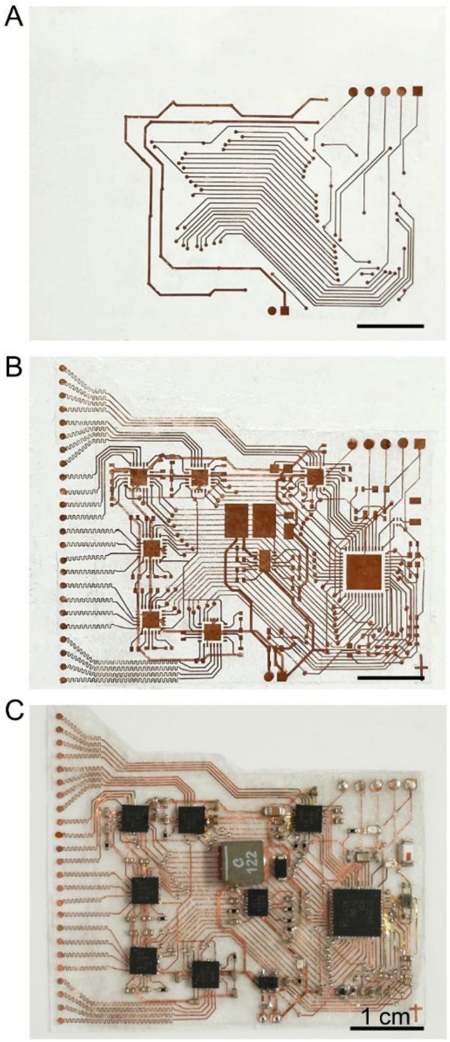

Fig. S3.

Photograph of the ES device fabrication process. (**A**) Bottom electric circuits. (**B**) Assembled top and bottom electric circuits before soldering. (**C**) Soldering of all through-holes and electric components.

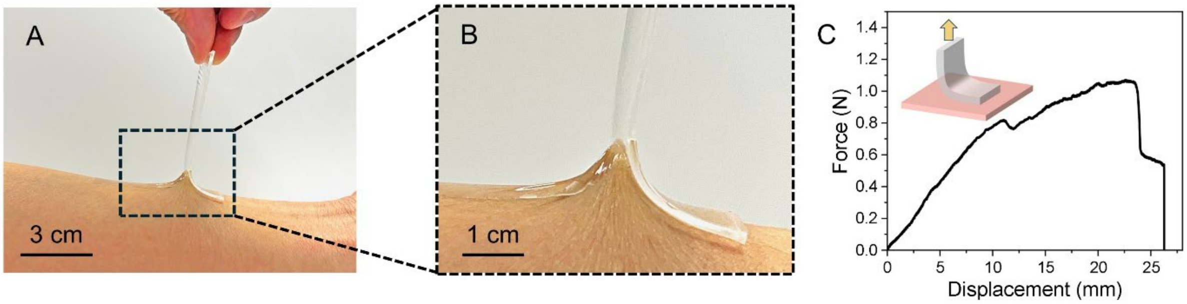

Fig. S4.

Adhesion between devices and the skin. (**A**) Photograph showing the peel-off process of a device adhered to the skin using medical-grade adhesive tape. (**B**) Magnified view of the interface between the tape and skin tissue during peeling. (**C**) Representative force-displacement curves from the peel-off test.

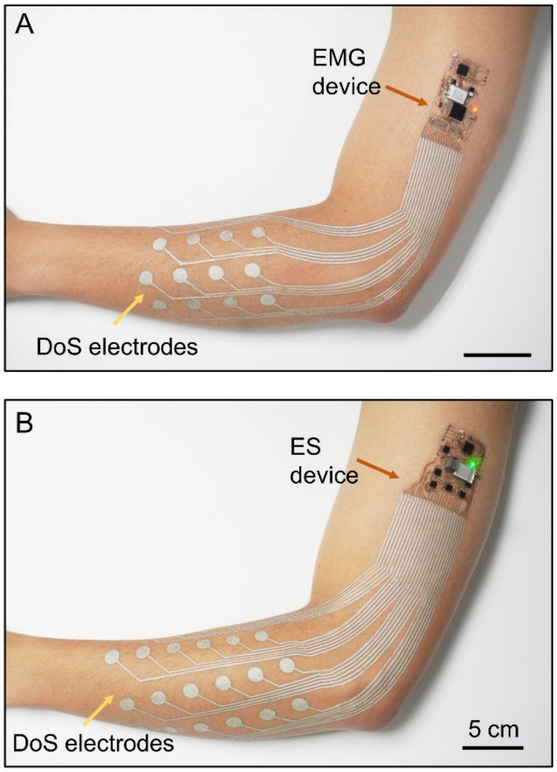

Fig. S5.

Photograph of the devices with DoS electrodes on the arm. (**A**) EMG detection device with DoS electrodes. (**B**) ES device with DoS electrodes.

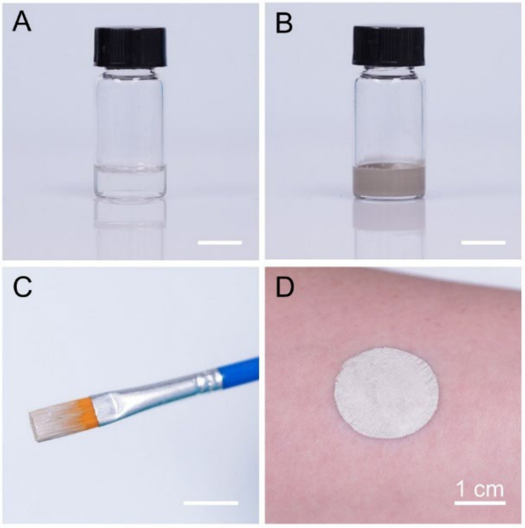

Fig. S6.

Fabrication of DoS electrodes. (**A**) Photograph of liquid band solvent. (**B**) Photograph of the DoS inks. (**C**) Brush loaded with DoS ink. (**D**) DoS electrode applied on skin.

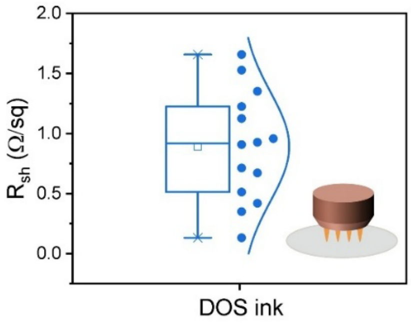

Fig. S7.

Electrical performance of DoS electrodes. The DoS ink exhibits a average sheet resistance of 0.89 Ω/sq, N=14.

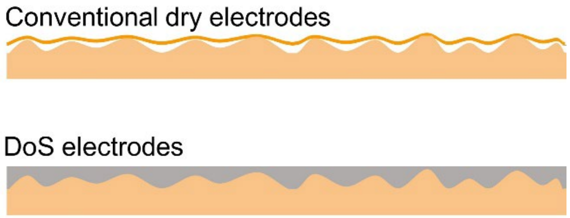

Fig. S8.

Schematic diagram of the interface between conventional dry electrodes and DoS electrodes on skin.

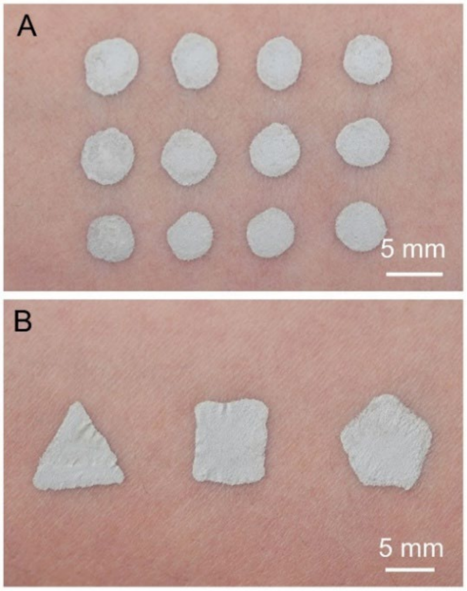

Fig. S9.

Photographs of the DoS electrodes on skin. (**A**) Points pattern. (**B**) Various shapes: triangle, square and pentagon.

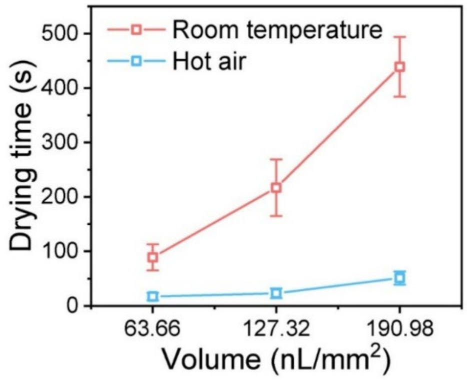

Fig. S10.

Variation in drying time at room temperature and under hot air conditions with different ink volumes for 10 mm-diameter DoS electrodes, N=5.

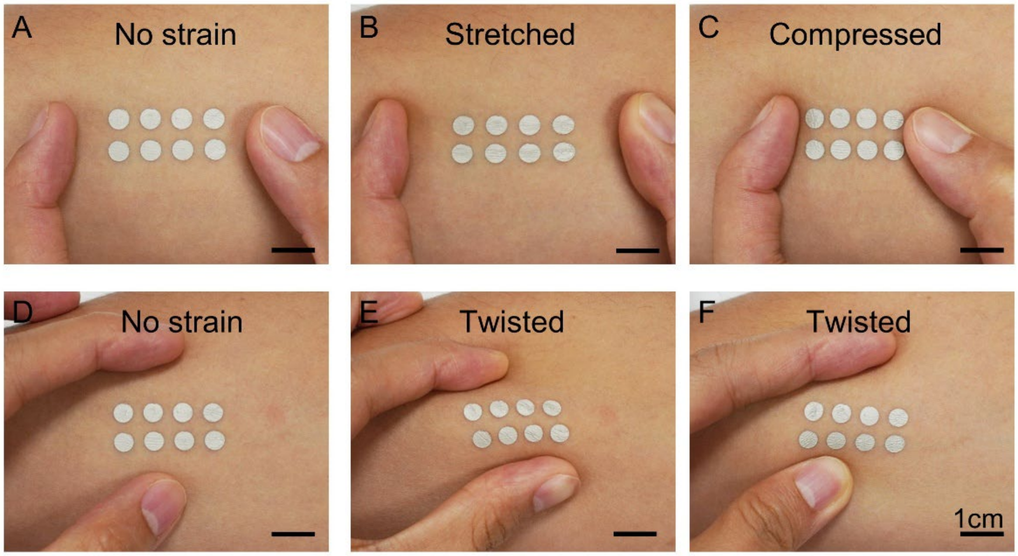

Fig. S11.

Photograph of 5 mm DoS electrodes deformed on skin. (**A**)-(**C**) Stretching of the DoS electrodes. (**D**)-(**F**) Twisting of the DoS electrodes.

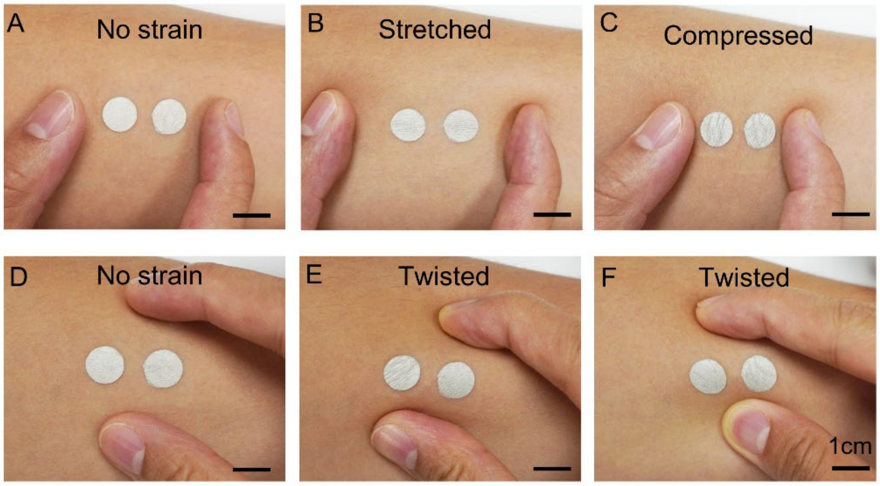

Fig. S12.

Photograph of 1 cm DoS electrodes deformed on skin. (**A**)-(**C**) Stretching of the DoS electrodes. (**D**)-(**F**) Twisting of the DoS electrodes.

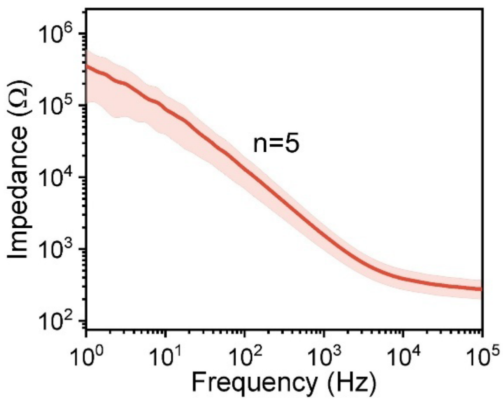

Fig. S13.

Statistical interface impedance between DoS electrodes and skin, N=5.

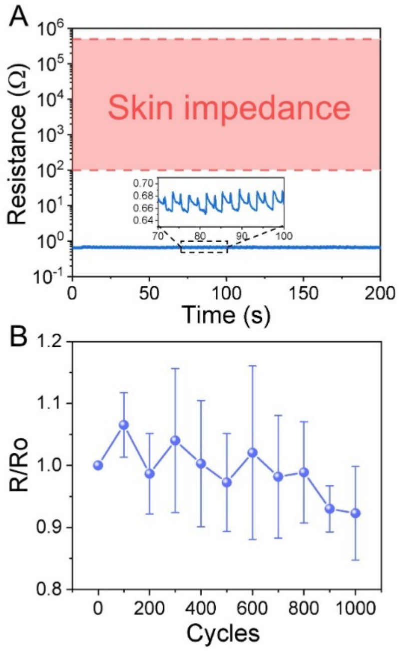

Fig. S14.

Characterization of DoS electrodes deformed on skin. (**A**) Resistance change of the DoS electrodes under tensile strain. (**B**) Relative resistance change over 1000 repeated stretching cycles, N=5.

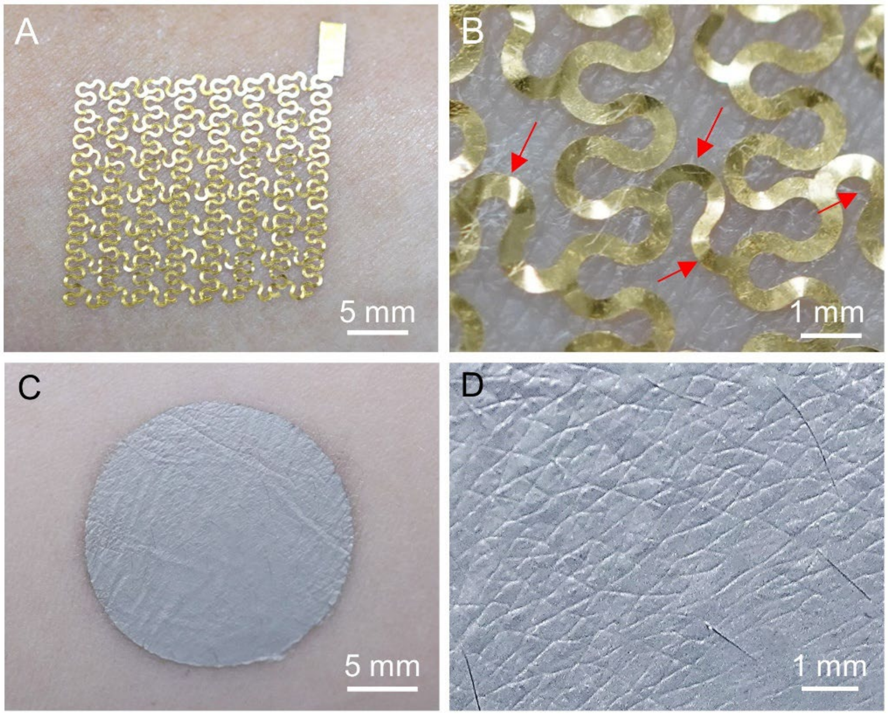

Fig. S15.

Photograph of the deformed electrodes on skin. (**A**) Au mesh electrodes after exercise. (**B**) Enlarged view of Au mesh electrode showing delamination and peeling. (**C**) DoS electrodes after exercise. (**D**) Enlarged view of the DoS electrodes showing conformal adhesion to the skin without noticeable delamination.

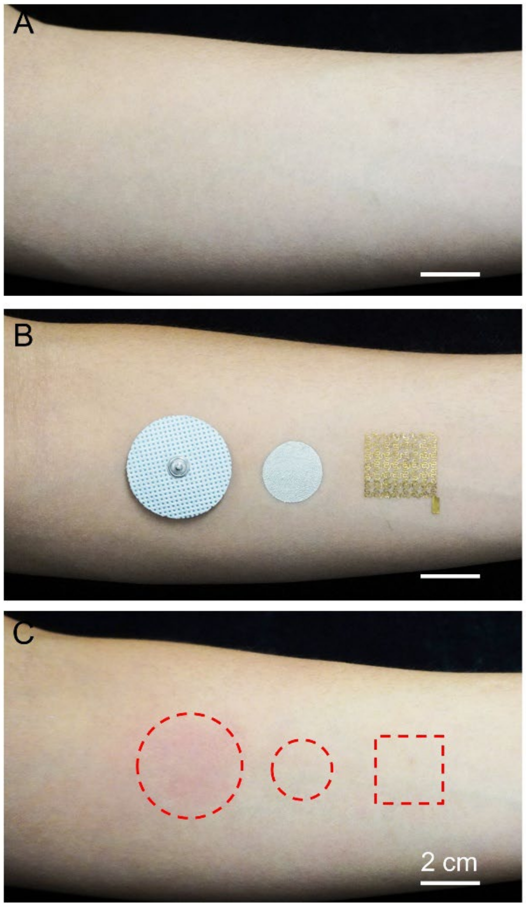

Fig. S16.

Photograph of the skin during the electrode-induced inflammation assessment. (**A**) Original and untreated skin. (**B**) Skin with electrodes applied. (**C**) Skin condition after wearing the electrodes continuously for 12 hours, showing the extent of any inflammation or irritation.

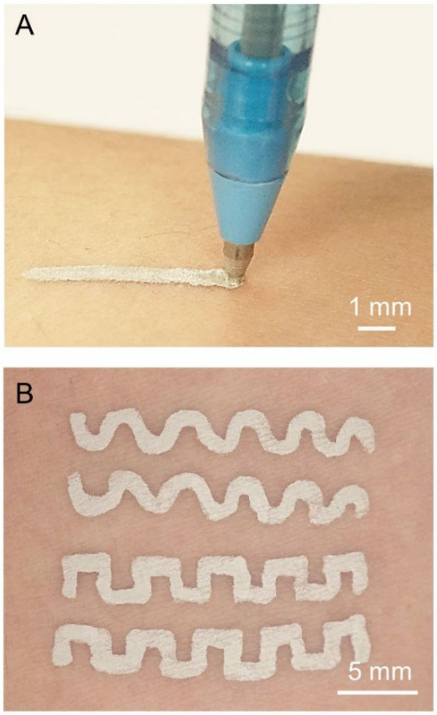

Fig. S17.

Photographs of drawing DoS electrodes on skin. (**A**) Schematic diagram of DoS ink application using a ballpen. (**B**) Conductive lines with different shapes.

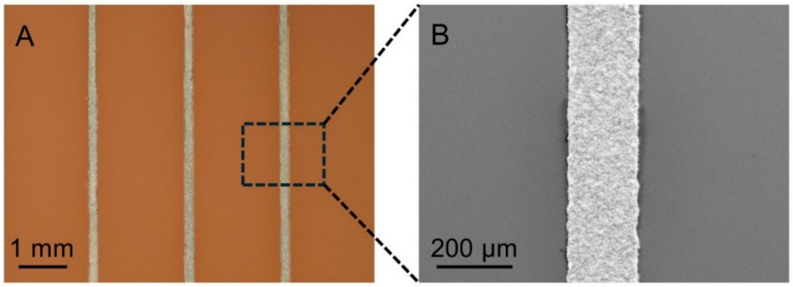

Fig. S18.
Linewidth and resolution of DoS lines. (**A**) DoS lines with a width of 200 µm drawn on PDMS substrates. (**B**) SEM image of an enlarged area of a DoS line.

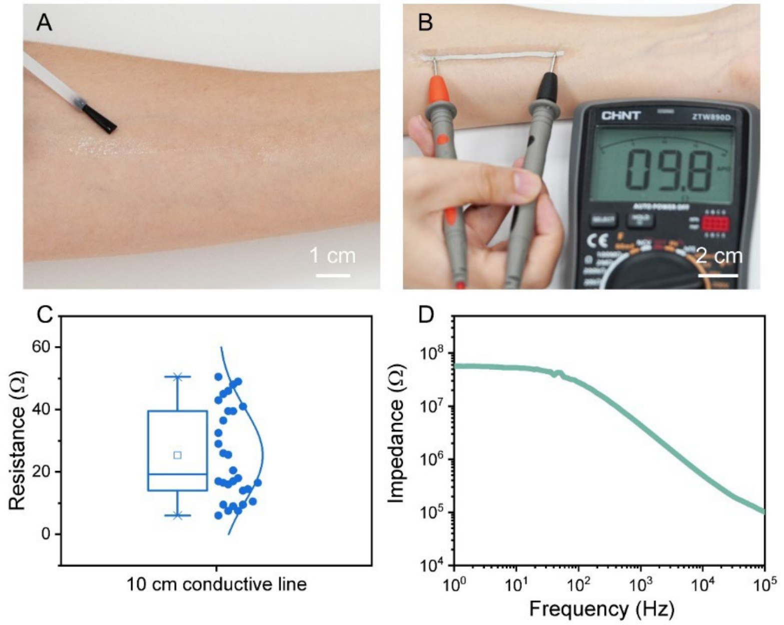

Fig. S19.

Characterization of conductive lines on skin. (**A**) A liquid bandage was applied to the skin, serving as an insulating substrate. (**B**) The DoS conductive line drawn on the insulating substrate exhibited a low resistance of 9.8 Ω. (**C**) Statistical distribution of resistance for conductive lines with a length of 10 cm and a width of 1 mm, N=30. (**D**) Skin impedance of the surface protected with thick glue and insulating materials.

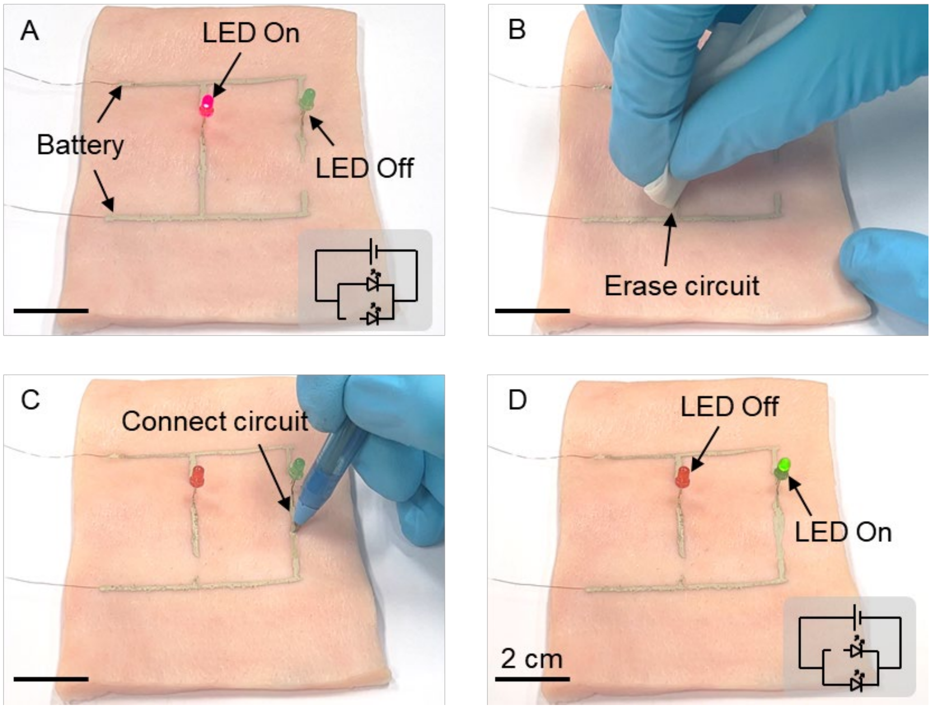

Fig. S20.
On-demand reconfiguration of DoS circuits. The circuit activating the red LED was selectively removed (**A**–**B**), and the target pathway was reconnected to activate the green LED (**C**–**D**).

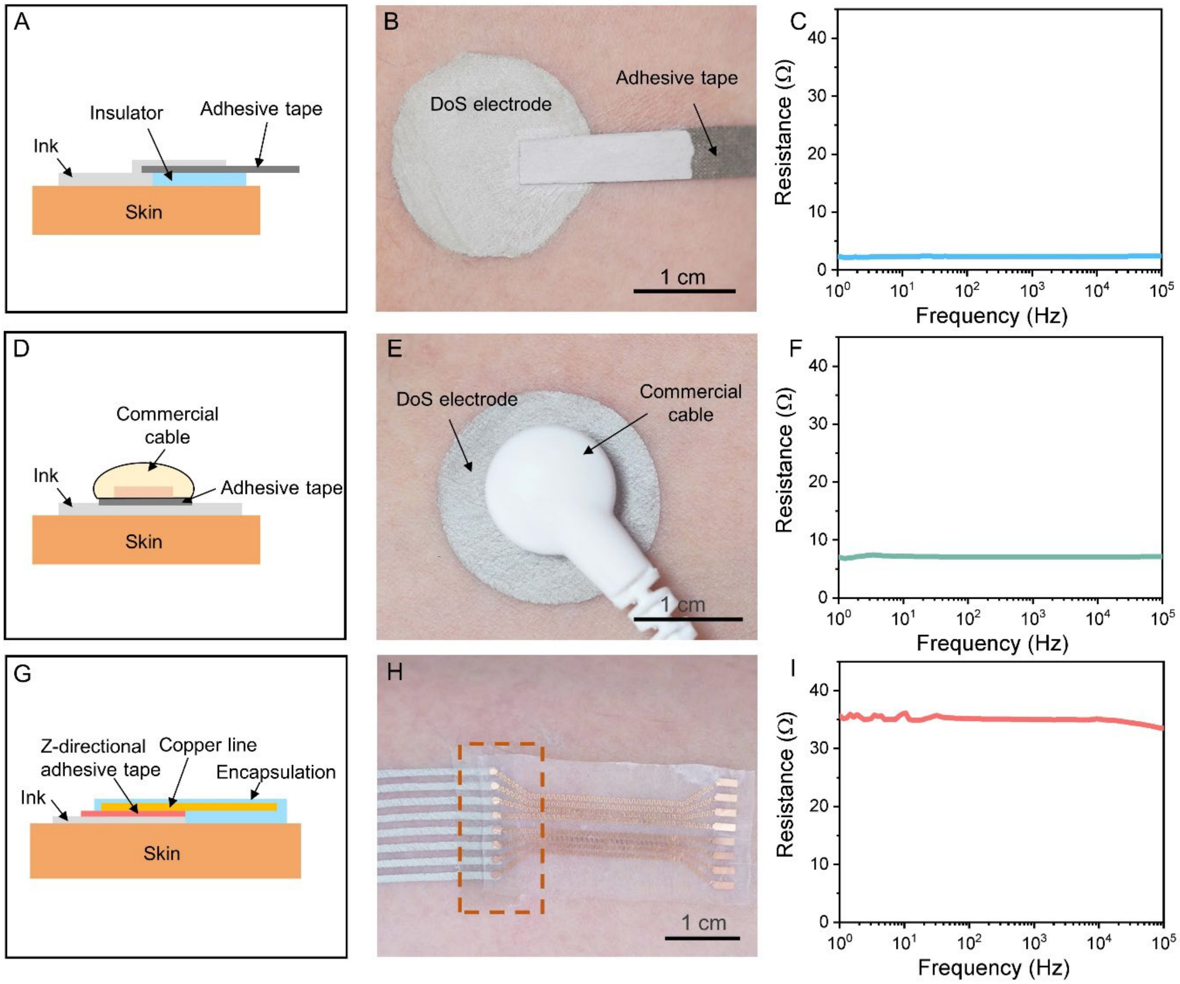

Fig. S21.

Characterization of the interconnection between DoS electrodes and external equipment. (**A**) Schematic diagram and (**B**) photograph of DoS electrodes connected with commercial double-sided conductive adhesive tape, and (**C**) corresponding resistance of the interconnection. (**D**) Schematic diagram and (**E**) the photograph of DoS electrodes connected with commercial cable using double-sided conductive adhesive tape, and (**F**) corresponding resistance of the interconnection. (**G**) Schematic diagram and (**H**) the photograph of DoS conductive lines connected with Z-directional conductive adhesive tape, and (**I**) corresponding resistance of the interconnection, which can be used as a conductive interface for a series of conductive electrodes.

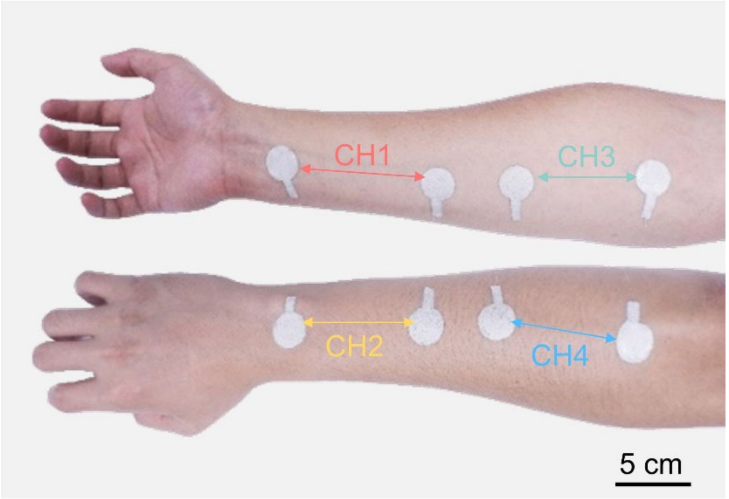

Fig. S22.

Photograph of DoS electrodes on skin used for collecting EMG signals for data acquisition in deep learning.

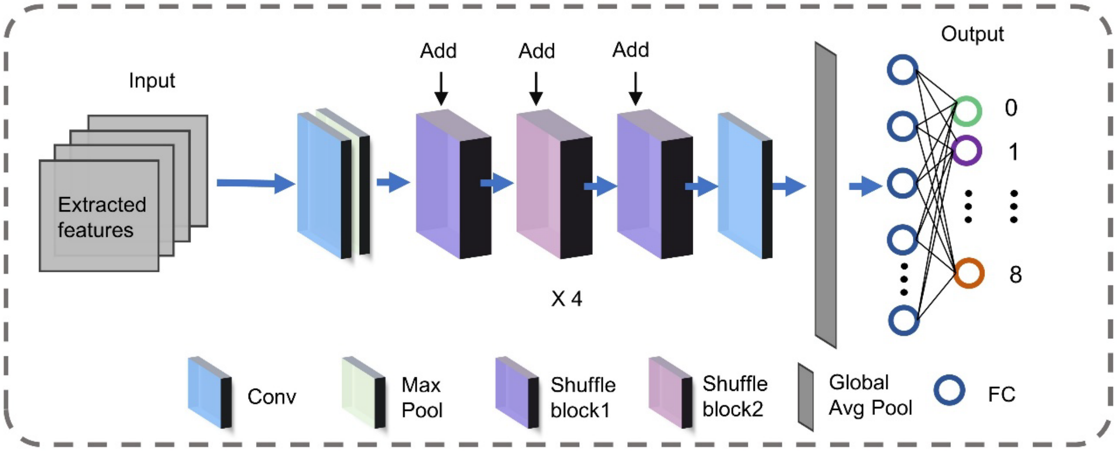

Fig. S23.
Architecture of the proposed deep learning model for gesture recognition.

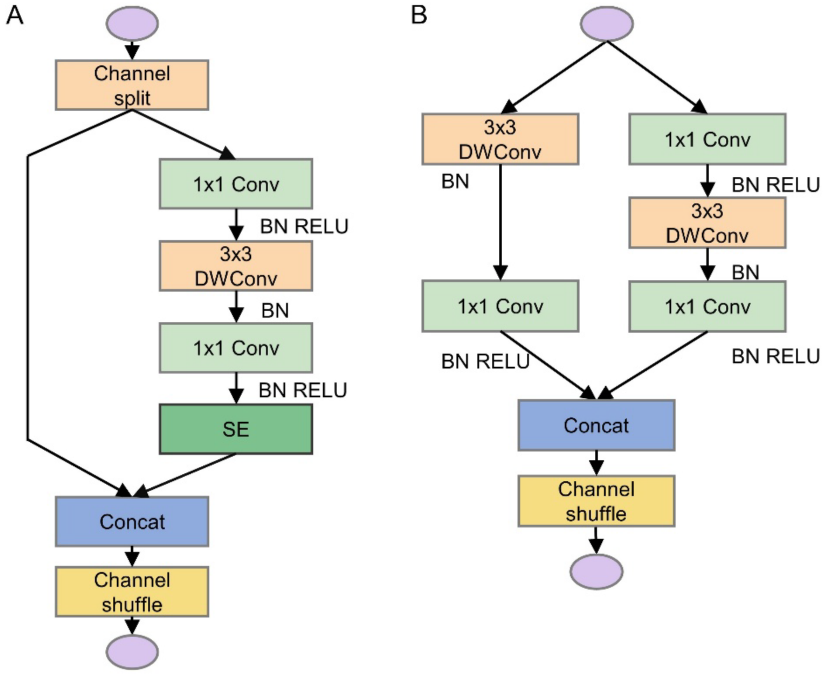

Fig. S24.

Detailed structure of shuffle block unit. (**A**) Structure of shuffle block 1. (**B**) Structure of shuffle block 2.

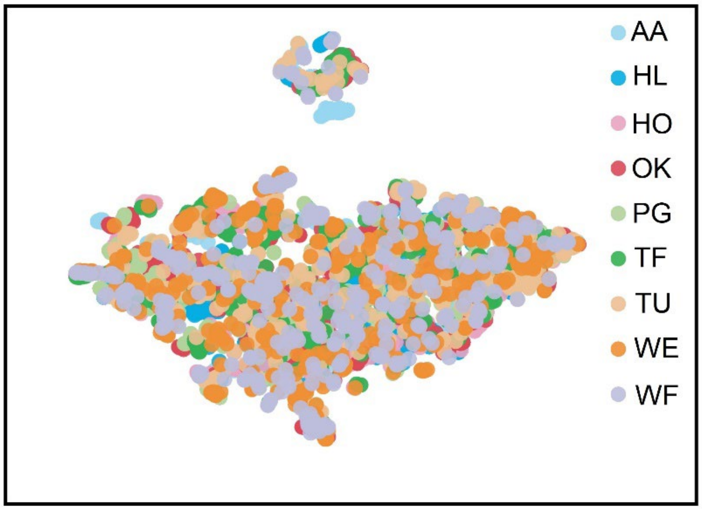

Fig. S25.

Scatter plot of original test data before classification.

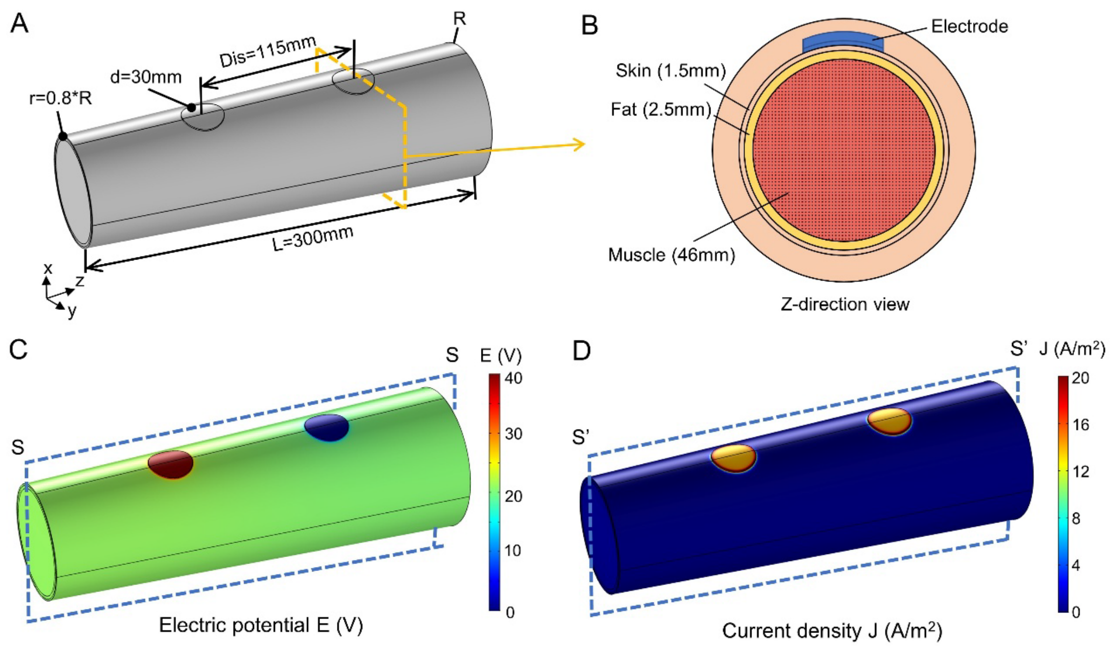

Fig. S26.

3D model of the forearm. (**A**) Geometry of the forearm model with two circular electrodes. (**B**) Cross-section of the forearm model at the electrode level, showing the thickness of different tissues. The dots in the muscle area represent the distribution of nerve bundles. (**C**) Electric potential and (**D**) current density distribution volume plot of the forearm under 40V stimulation.

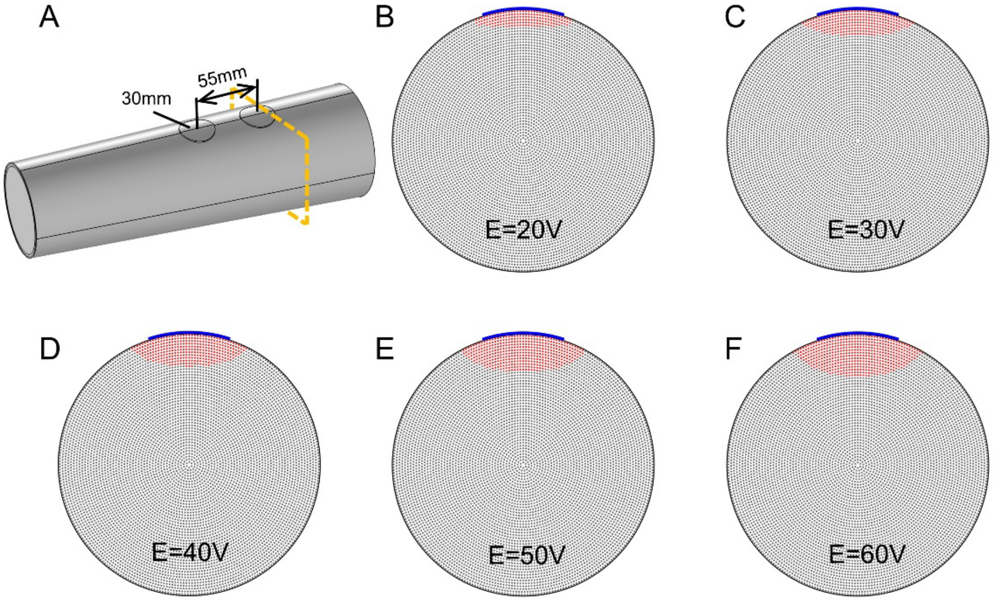

Fig. S27.

Simulation study of the forearm model featuring two circular electrodes. (**A**) Geometry of the forearm model, where each electrode has a diameter of 30 mm and they spaced 55 mm apart. Distribution of activated nerve bundles under (**B**) E = 20V, (**C**) E = 30V, (**D**) E = 40V, (**E**) E = 50V, and (**F**) E = 60V.

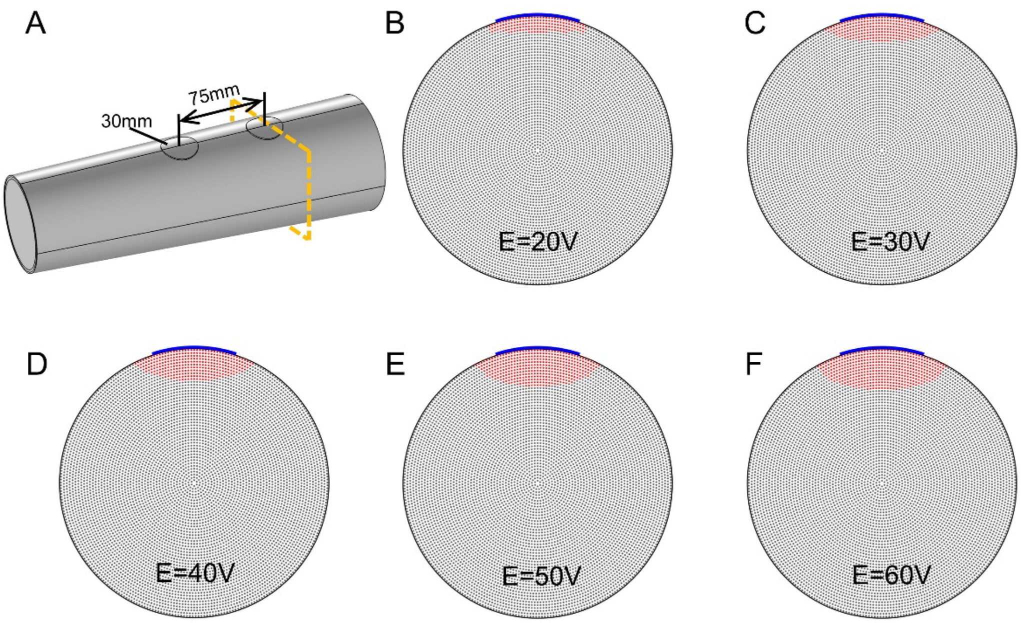

Fig. S28.

Simulation study of the forearm model featuring two circular electrodes. (**A**) Geometry of the forearm model, where each electrode has a diameter of 30 mm and they spaced 75 mm apart. Distribution of activated nerve bundles under (**B**) E = 20V, (**C**) E = 30V, (**D**) E = 40V, (**E**) E = 50V, and (**F**) E = 60V.

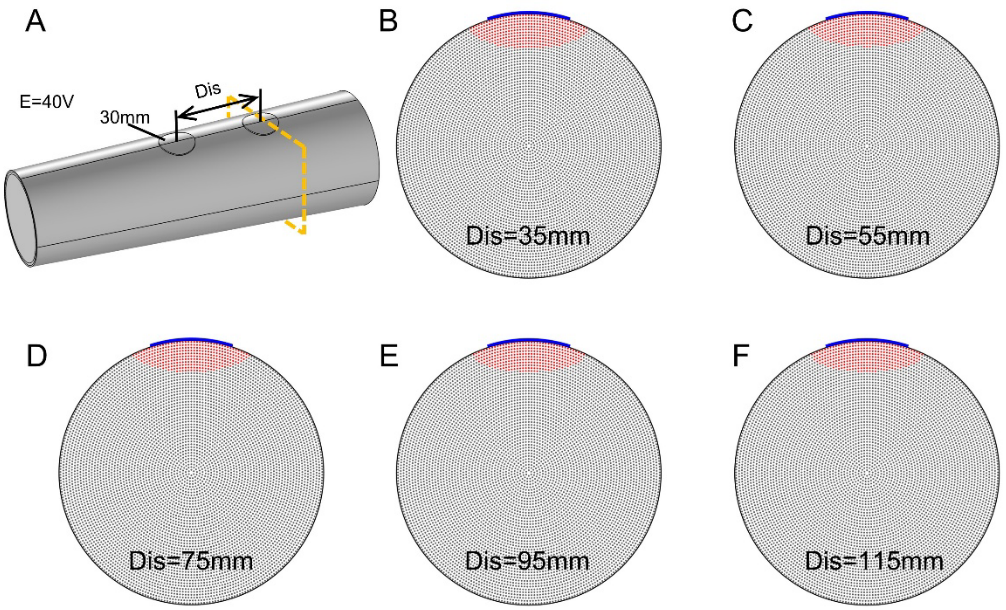

Fig. S29.

Simulation study of the forearm model featuring two circular electrodes. (**A**) Geometry of the forearm model, where each electrode has a diameter of 30 mm, under E = 40V. Distribution of activated nerve bundles when the electrodes are spaced: (**B**) 35 mm apart, (**C**) 55 mm apart, (**D**) 75 mm apart, (**E**) 95 mm apart, and (**F**) 115 mm apart.

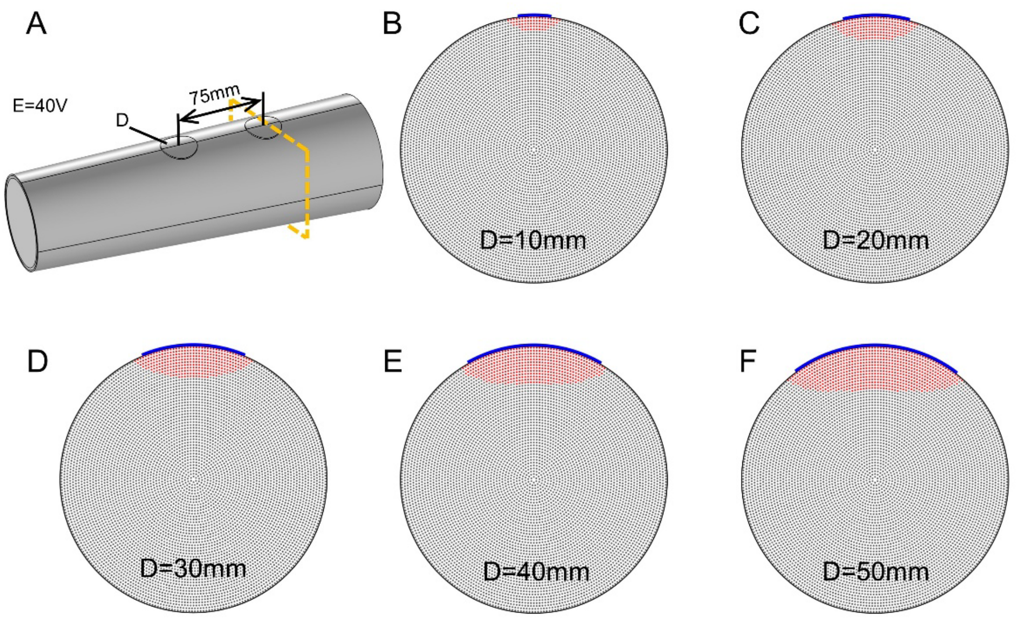

Fig. S30.

Simulation study of the forearm model featuring two circular electrodes. (**A**) Geometry of the forearm model, where two electrodes are spaced 75 mm apart, under E = 40V. Distribution of activated nerve bundles when the electrode diameter D is (**B**) 10 mm, (**C**) 20 mm, (**D**) 30 mm, (**E**) 40 mm, and (**F**) 50 mm.

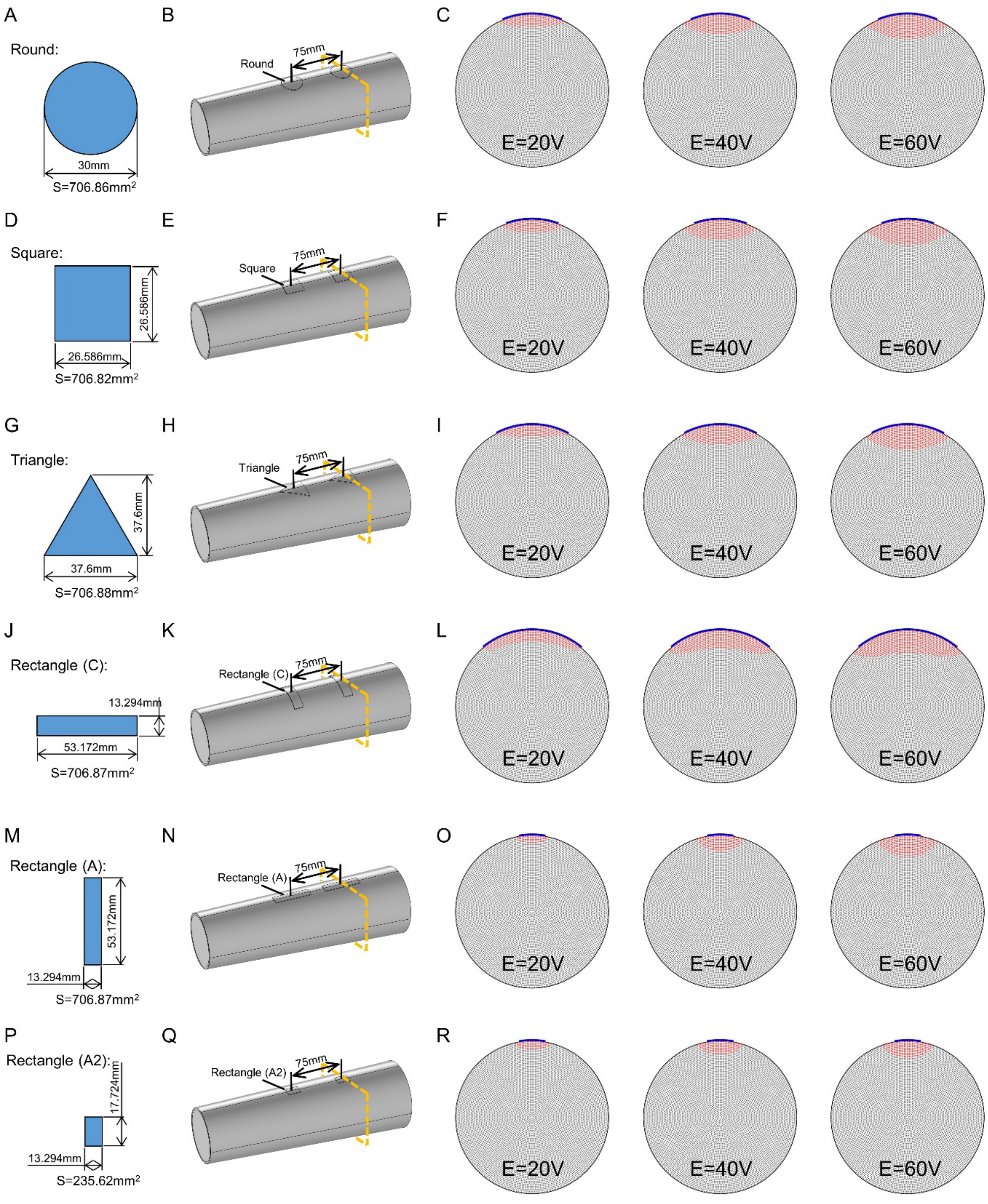

Fig. S31.

Simulation study of the forearm model featuring two electrodes spaced 75 mm apart under E = 20-60V. Electrode shapes are (**A**)-(**C**) round, (**D**)-(**F**) square, (**G**)-(**I**) triangle, (**J**)-(**L**) rectangle with long sides along the circumference, (**M**)-(**O**) rectangle with long side along axis, and (**P**)-(**R**) rectangle with small area.

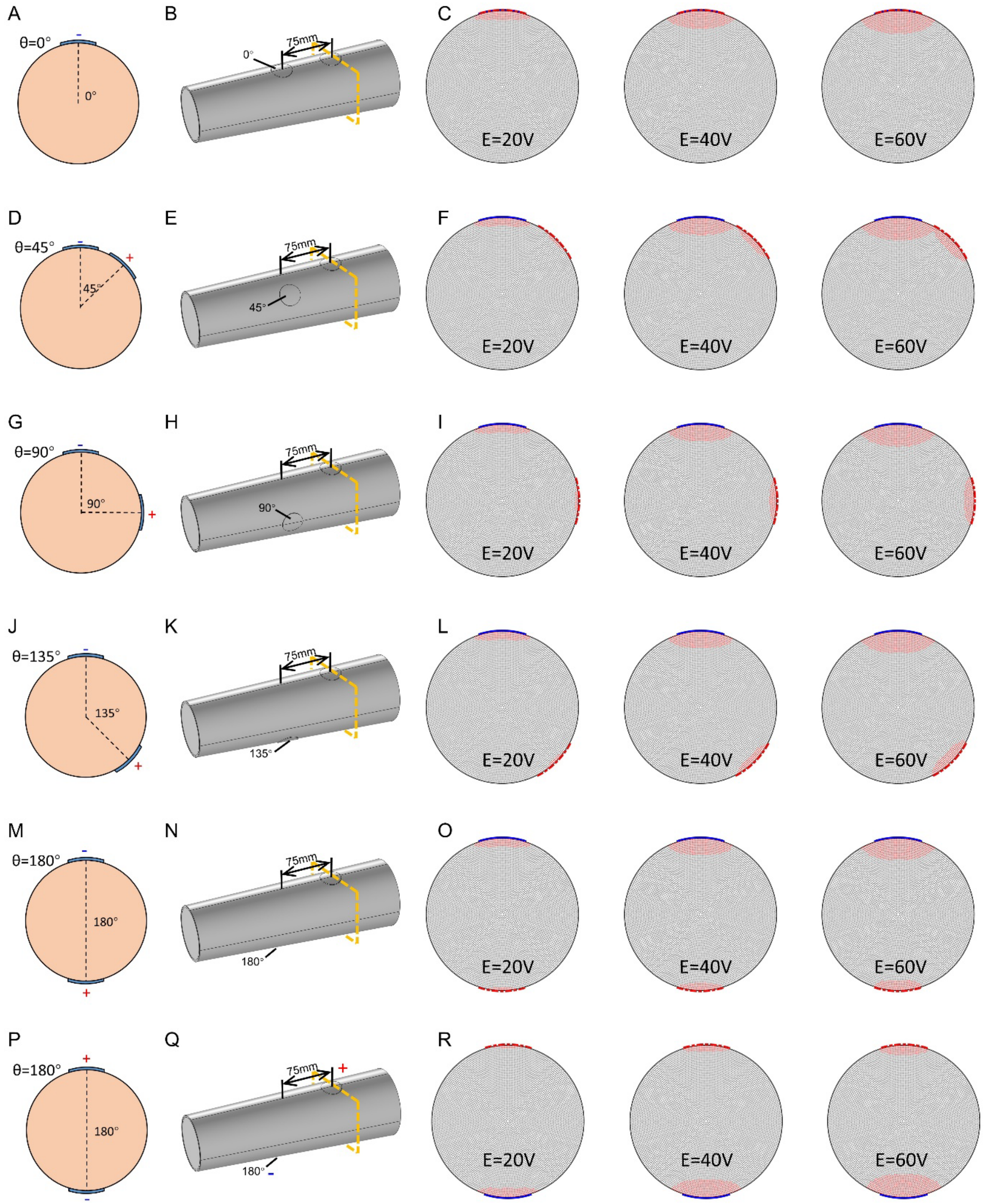

Fig. S32.

Simulation study of the forearm model featuring two circular electrodes spaced 75 mm apart under E = 20-60V. The angle between the two electrodes is (**A**)-(**C**) 0°, (**D**)-(**F**) 45°, (**G**)-(**I**) 90°, (**J**)-(**L**) 135°, (**M**)-(**O**) 180°, and (**P**)-(**R**) 180° with the positive and negative poles reversed.

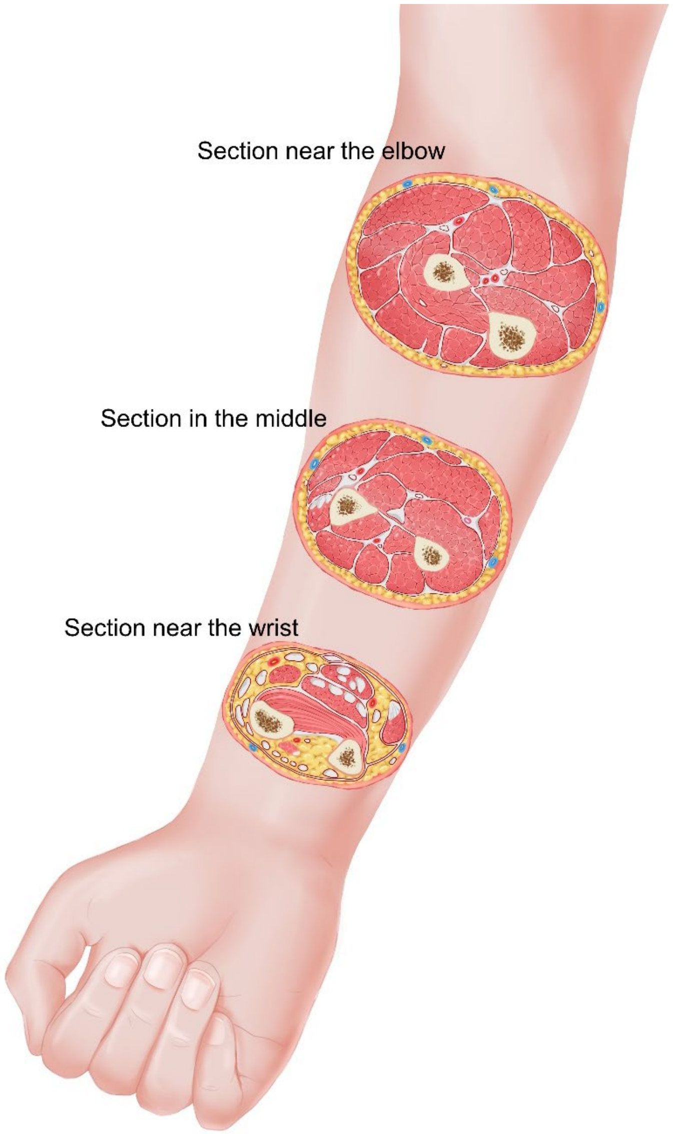

Fig. S33.
Anatomical cross-section of forearm muscle distribution.

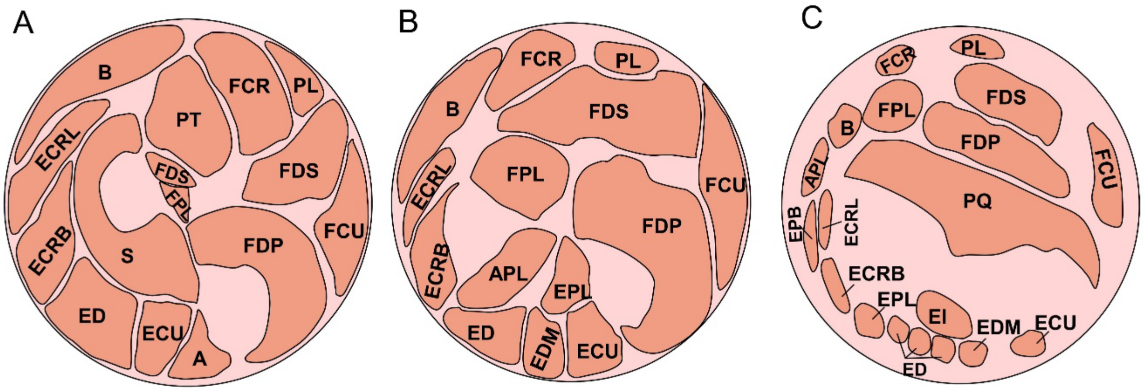

D

	Wrist	Thumb	Index finger	Middle finger	Ring finger	Little finger
**Flexion**	**FCU:** Flexor carpi ulnaris; **FCR:** Flexor carpi radialis; **PL:** Palmaris longus;	**FPL:** Flexor pollicis longus;	**FDS:** Flexor digitorum superficialis; **FDP:** Flexor digitorum profundus			
**Extension**	**ECRB:** Extensor carpi radialis brevis; **ECRL:** Extensor carpi radialis longus; **ECU:** Extensor carpi ulnaris	**APL:** Abductor pollicis longus **EPL:** Extensor pollicis longus; **EPB:** Extensor pollicis brevis	**EI:** Extensor indicis<br>**ED:** Extensor digitorum	**ED:** Extensor digitorum		**EDM:** Extensor digiti minimi<br>**ED:** Extensor digitorum

B: Brachioradialis **PQ:** Pronator quadratus

Fig. S34.

Forearm muscle distribution. Cross-section of forearm muscle distribution in the (**A**) section near the elbow, (**B**) section in the middle, (**C**) section near the wrist. (**D**) muscles associated with specific movements of particular joints.

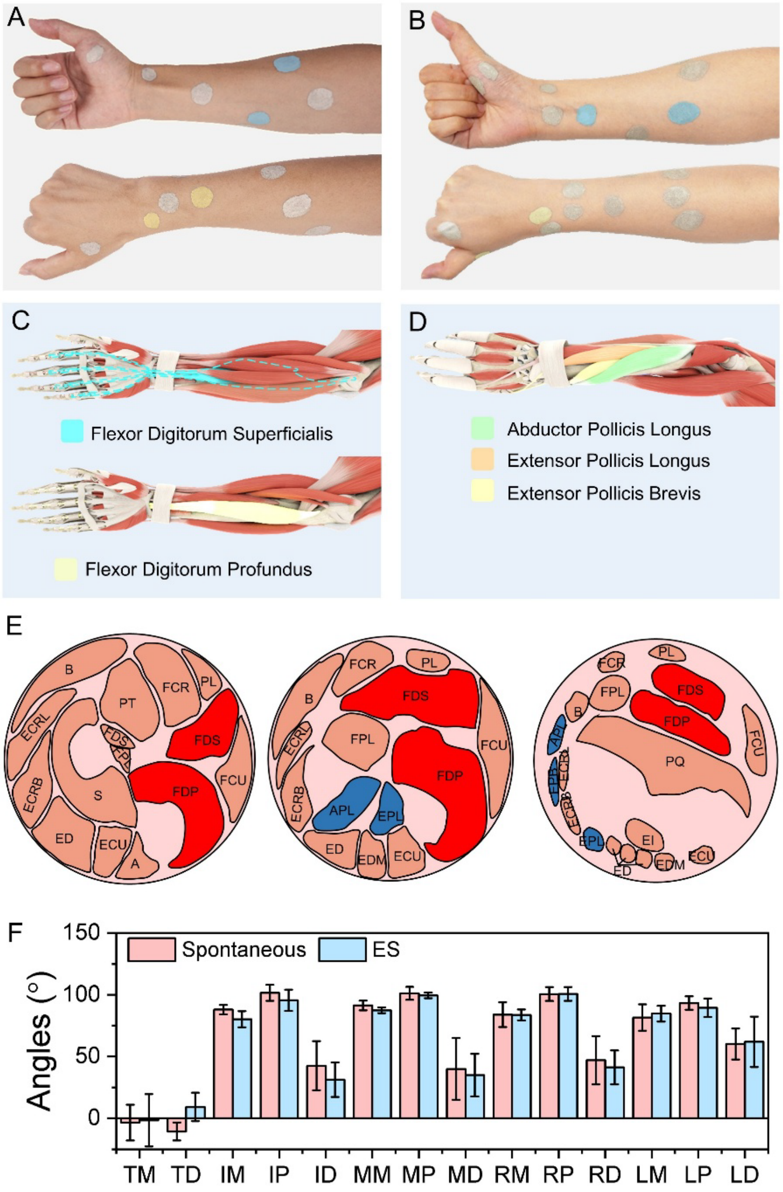

Fig. S35.

Activated forearm muscles for the gesture of thumb up. (**A**)-(**B**) Photographs of the DoS electrodes on two participants' forearms. The electrodes used for activating flexion of four fingers and extension of thumb are marked with blue and yellow shading, respectively. (**C**) Muscles related to flexion of four fingers. (**D**) Muscles related to extension of the thumb. (**E**) Cross-section of activated forearm muscles. (**F**) Bending angles of different finger joints in spontaneous and ES-induced thumb up gestures.

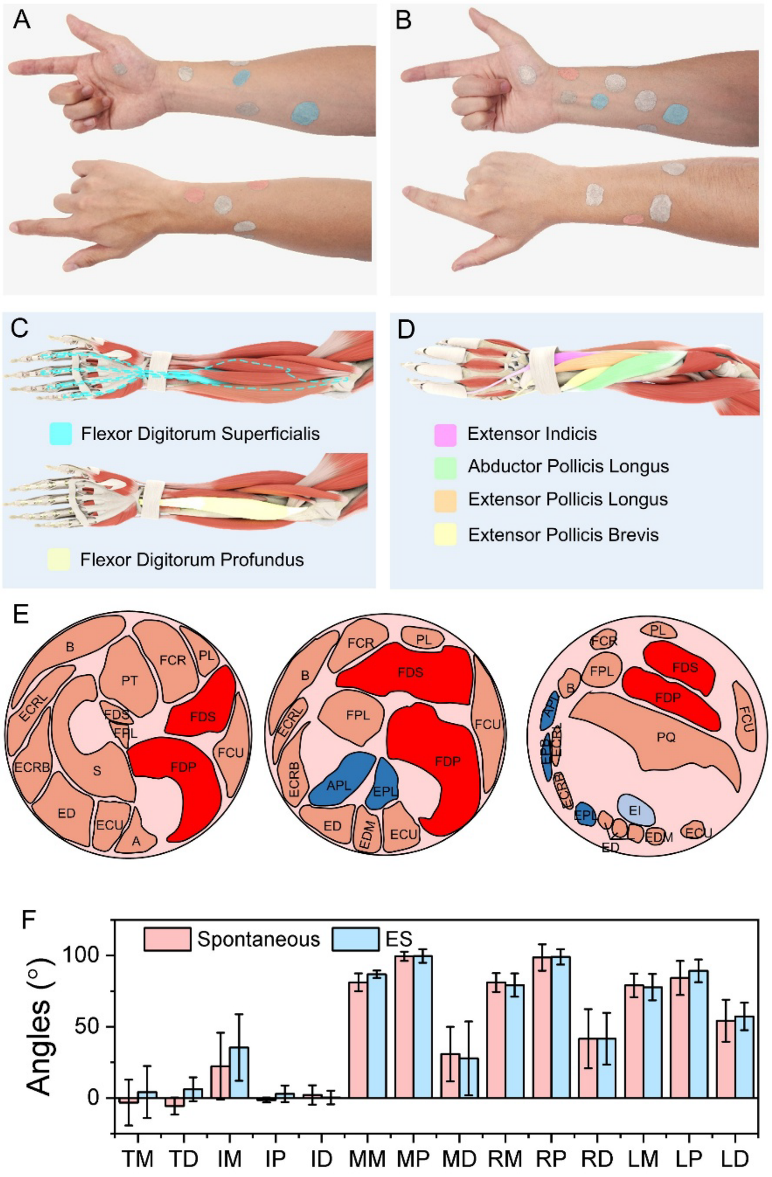

Fig. S36.

Activated forearm muscles for the gesture of acute angle. (**A**)-(**B**) Photographs of the DoS electrodes on two participants' forearms. The electrodes used for activating flexion of middle, ring, and little fingers, as well as extension of the thumb and index finger, are marked with blue and pink shading, respectively. (**C**) Muscles related to flexion of middle, ring, and little fingers. (**D**) Muscles related to the extension of the thumb and index finger. (**E**) Cross-section of activated forearm muscles. (**F**) Bending angles of different finger joints in spontaneous and ES-induced acute angle gestures, N=5.

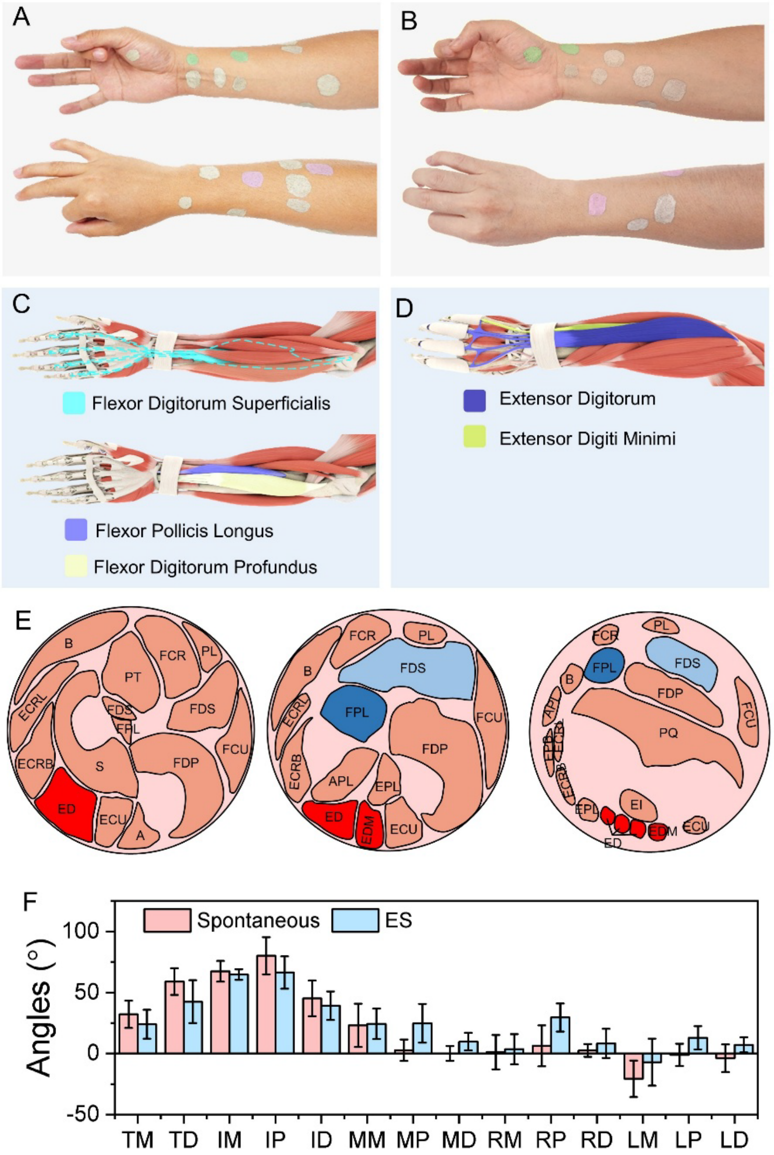

Fig. S37.

Activated forearm muscles for the gesture of OK. (**A**)-(**B**) Photographs of the DoS electrodes on two participants' forearms. The electrodes used for activating extension of the middle, ring, and little fingers, as well as the flexion of thumb and index finger are marked with purple and green shading, respectively. (**C**) Muscles related to the flexion of thumb and index finger. (**D**) Muscles related to the extension of middle, ring, and little fingers. (**E**) Cross-section of activated forearm muscles. (**F**) Bending angles of different finger joints in spontaneous and ES-induced OK gestures, N=5.

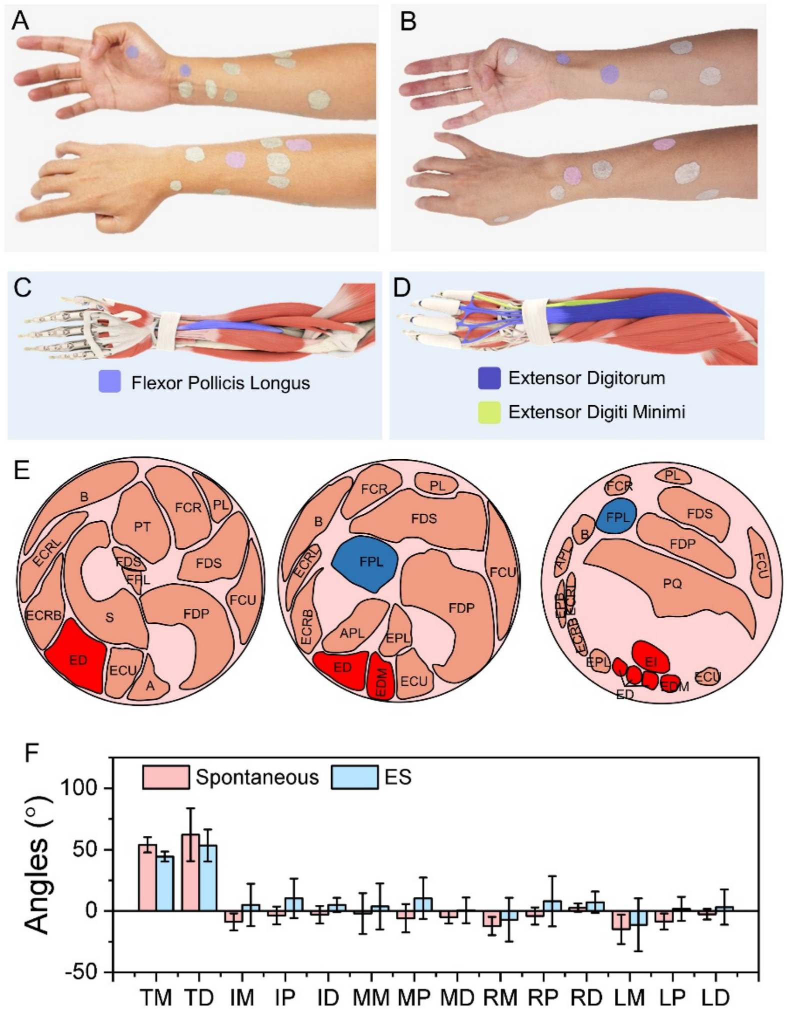

Fig. S38.

Activated forearm muscles for the gesture of thumb flexed. (**A**)-(**B**) Photographs of the DoS electrodes on two participants' forearms. The electrodes used for activating flexion of the thumb and extension of the index, middle, ring, and little fingers are marked with dark purple and light purple shading, respectively. (**C**) Muscles related to the flexion of thumb. (**D**) Muscles related to the extension of index, middle, ring, and little fingers. (**E**) Cross-section of activated forearm muscles. (**F**) Bending angles of different finger joints in spontaneous and ES-induced thumb flexed gestures, N=5.

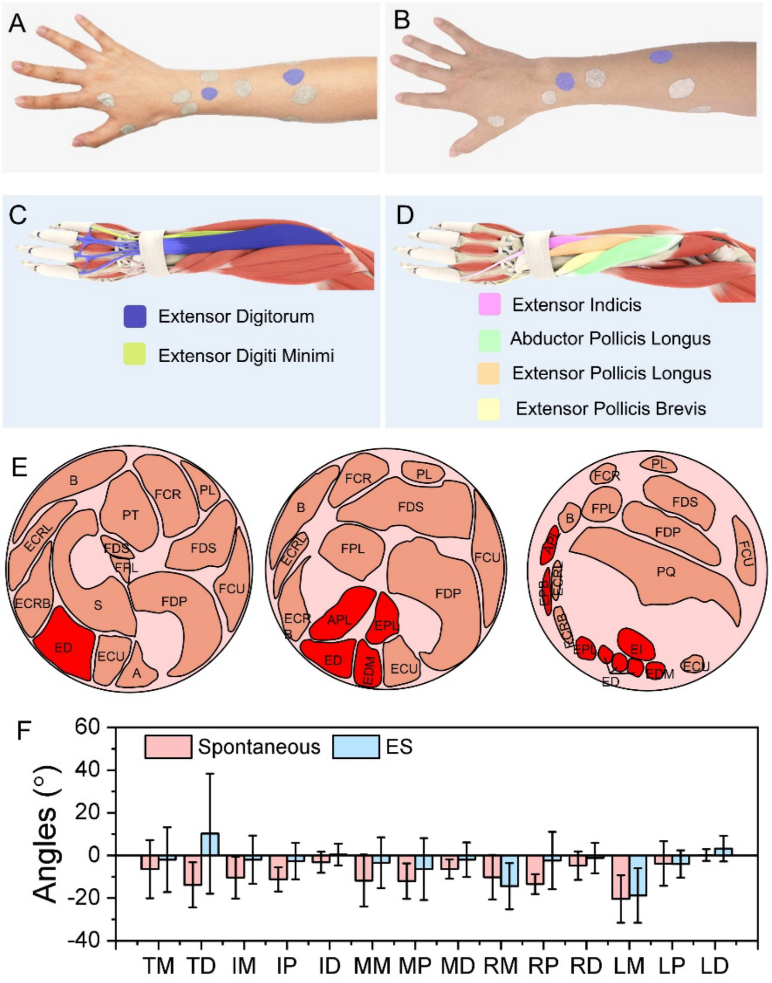

Fig. S39.

Activated forearm muscles for the gesture of hand open. (**A**)-(**B**) Photographs of the DoS electrodes on two participants' forearms. The electrodes used for activating extension of all fingers are marked with dark purple shading. Muscles related to extension all fingers in (**C**) the deep layer and (**D**) the superficial layer. (**E**) Cross-section of activated forearm muscles. (**F**) Bending angles of different finger joints in spontaneous and ES-induced hand open gestures, N=5.

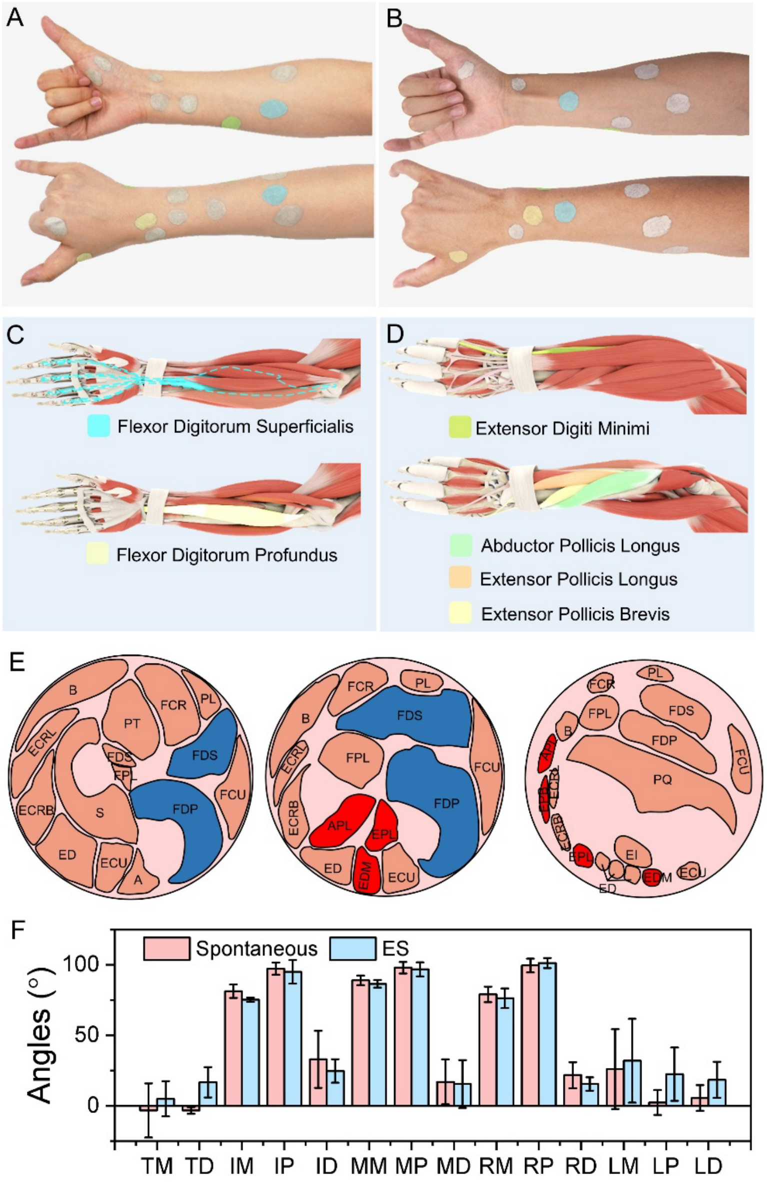

Fig. S40.

Activated forearm muscles for the gesture of hand loose. (**A**)-(**B**) Photographs of the DoS electrodes on two participants' forearms. The electrodes used for activating extension of the little finger, flexion of the index, middle and ring fingers, and extension of thumb are marked with green, light blue and yellow shading, respectively. Muscles related to (**C**) flexion and (**D**) extension. (**E**) Cross-section of activated forearm muscles. (**F**) Bending angles of different finger joints in spontaneous and ES-induced hand loose gestures, N=5.

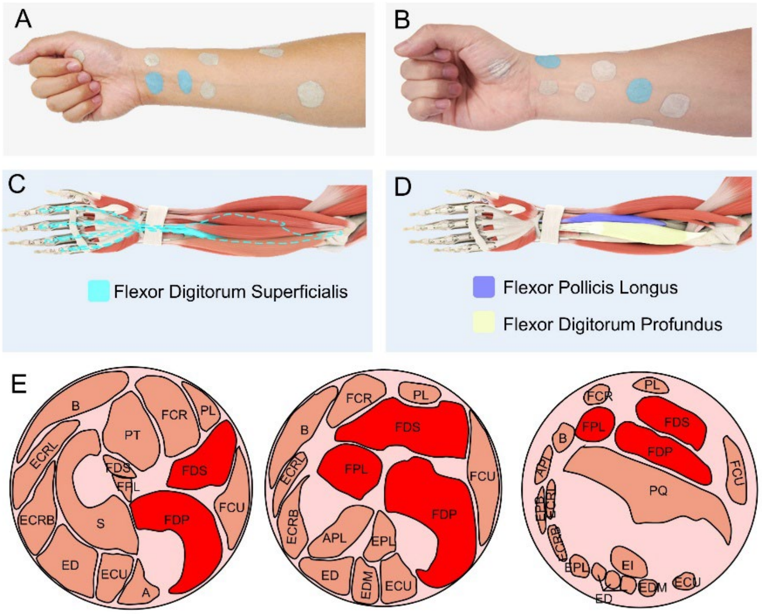

Fig. S41.

Activated forearm muscles for the gesture of power grip. (**A**)-(**B**) Photographs of the DoS electrodes on two participants' forearms. The electrodes used for activating flexion of all fingers are marked with light blue shading. Muscles related to flexion of all fingers in (**C**) the superficial layer and (**D**) the deep layer. (**E**) Cross-section of activated forearm muscles. (**F**) Bending angles of different finger joints in spontaneous and ES-induced power grip gestures.

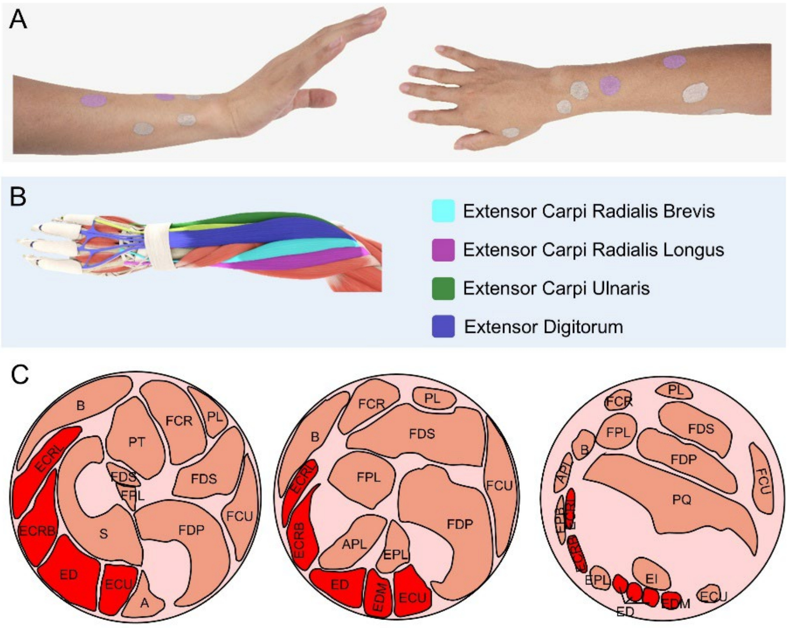

Fig. S42.

Activated forearm muscles for the gesture of wrist extension. (**A**) Photographs of the DoS electrodes on a participant' s forearm, with the electrodes used for activating wrist extension marked with dark purple shading. (**B**) Muscles related to wrist extension. (**C**) Cross-section of activated forearm muscles.

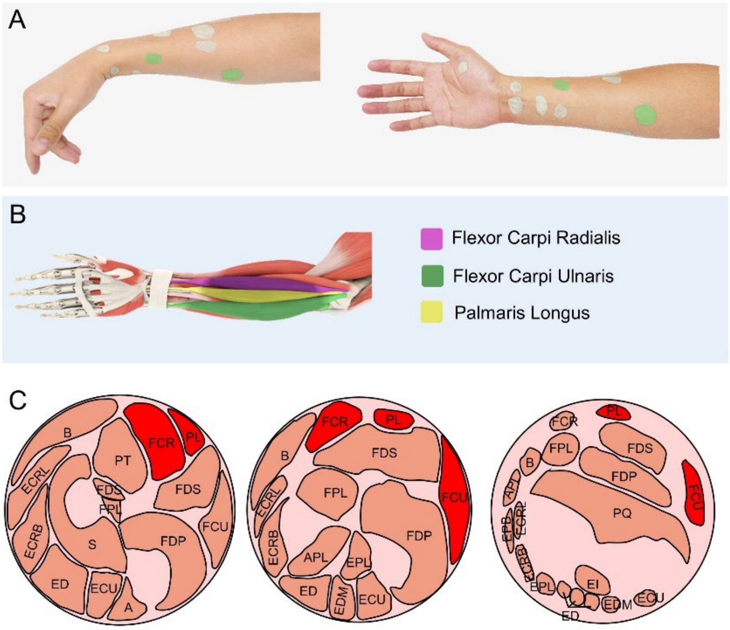

Fig. S43.

Activated forearm muscles for the gesture of wrist flexion. (**A**) Photographs of the DoS electrodes on a participant's forearm, with the electrodes used for activating wrist flexion marked with green shading. (**B**) Muscles related to wrist flexion. (**C**) Cross-section of activated forearm muscles.

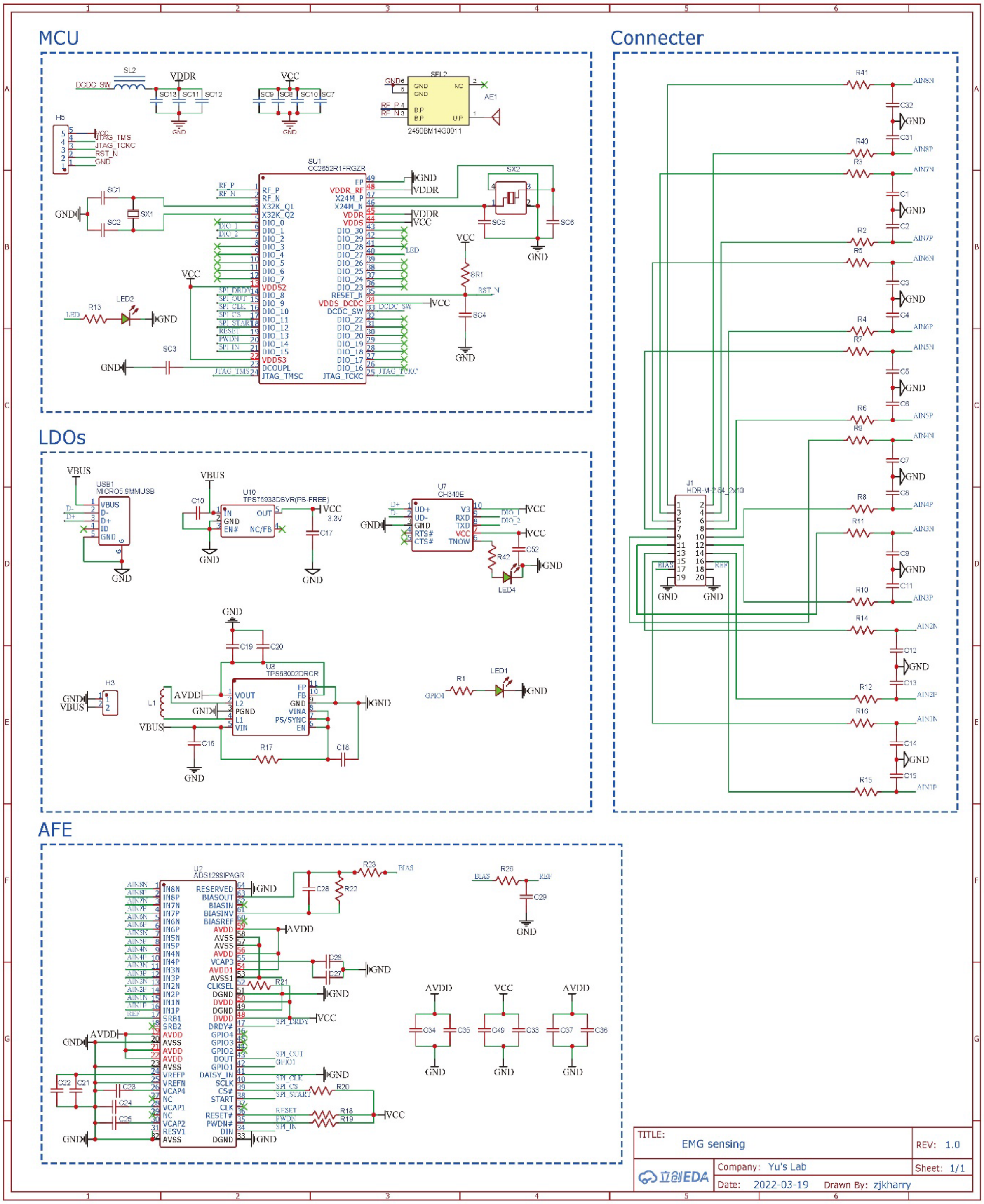

Fig. S44.

The circuits diagram of EMG circuits.

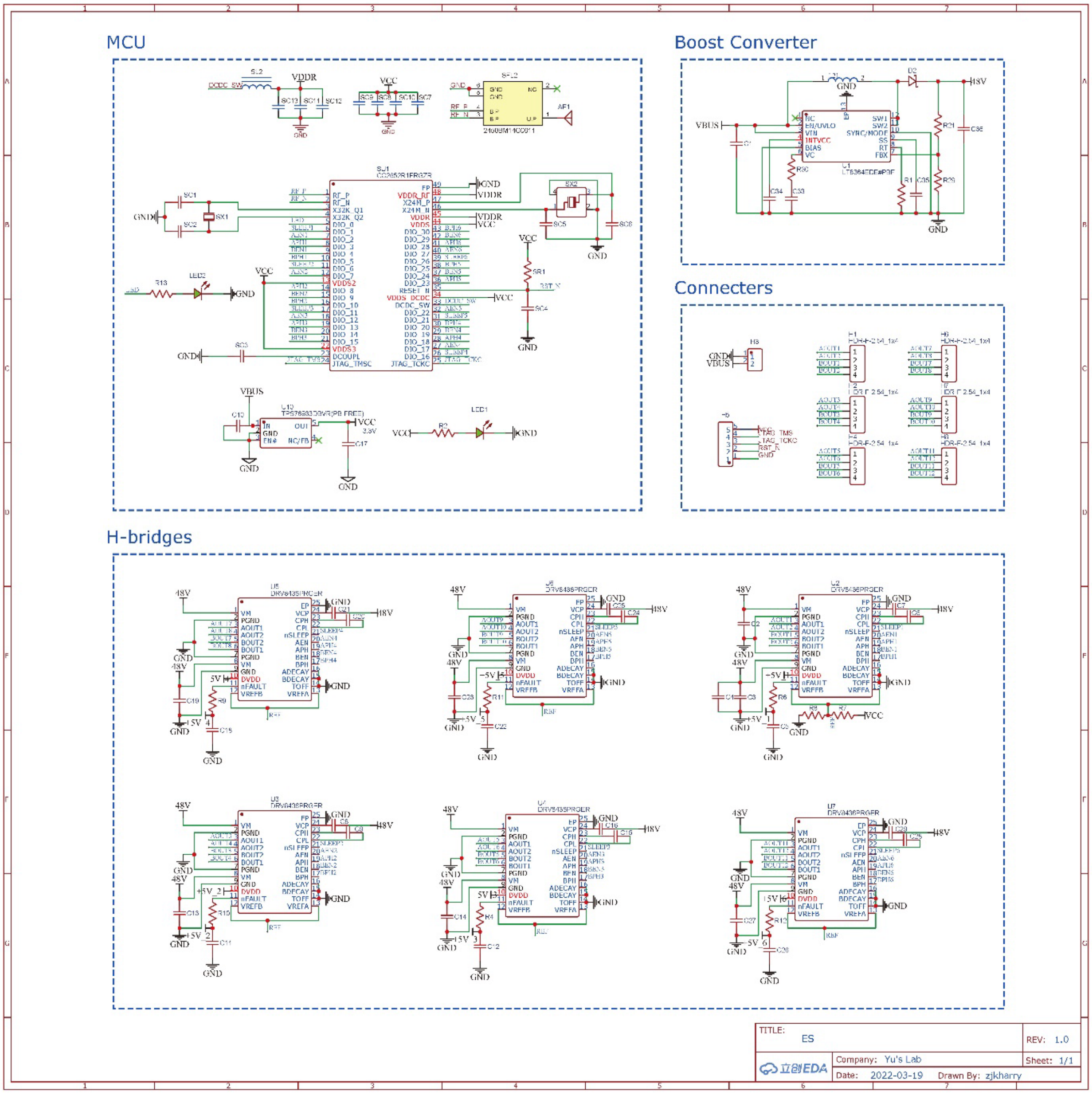

Fig. S45.

The circuits diagram of ES circuits.

Movie S1.
The process of handwriting customized electrodes.

Movie S2.
The process of handwriting customized the electrode lead.

Movie S3.
Follow motion experiment: Lifting an object with arm flexion.

Movie S4.
Two-hand coordination to lift a heavy object.

REFERENCES

1. Y. Huang, K. Yao, Q. Zhang, X. Huang, Z. Chen, Y. Zhou, X. Yu, Bioelectronics for electrical stimulation: Materials, devices and biomedical applications. *Chem. Soc. Rev.* **53**, 8632–8712 (2024).

2. R. D. Wilson, S. J. Page, M. Delahanty, J. S. Knutson, D. D. Gunzler, L. R. Sheffler, J. Chae, Upper-limb recovery after stroke: A randomized controlled trial comparing EMG-triggered, cyclic, and sensory electrical stimulation. *Neurorehabil. Neural Repair* **30**, 978–987 (2016).

3. M. Sun, C. Smith, D. Howard, L. Kenney, H. Luckie, K. Waring, P. Taylor, E. Merson, S. Finn, FES-UPP: A flexible functional electrical stimulation system to support upper limb functional activity practice. *Front. Neurosci.* **12**, 449 (2018).

4. N. M. Malešević, L. Z. P. Maneski, V. Ilić, N. Jorgovanović, G. Bijelić, T. Keller, D. B. Popović, A multi-pad electrode based functional electrical stimulation system for restoration of grasp. *J. Neuroeng. Rehabil.* **9**, 66 (2012).

5. K. Lee, Balance training with electromyogram-triggered functional electrical stimulation in the rehabilitation of stroke patients. *Brain Sci.* **10**, 80 (2020).

6. B. J. Forrester, J. S. Petrofsky, Effect of electrode size, shape, and placement during electrical stimulation. *J. Appl. Res.* **4**, 346–354 (2004).

7. L. Tian, B. Zimmerman, A. Akhtar, K. J. Yu, M. Moore, J. Wu, R. J. Larsen, J. W. Lee, J. Li, Y. Liu, B. Metzger, S. Qu, X. Guo, K. E. Mathewson, J. A. Fan, J. Cornman, M. Fatina, Z. Xie, Y. Ma, J. Zhang, Y. Zhang, F. Dolcos, M. Fabiani, G. Gratton, T. Bretl, L. J. Hargrove, P. V. Braun, Y. Huang, J. A. Rogers, Large-area MRI-compatible epidermal electronic interfaces for prosthetic control and cognitive monitoring. *Nat. Biomed. Eng.* **3**, 194–205 (2019).

8. J. Nishida, K. Suzuki, *paper presented at the Proceedings of the 2017 CHI Conference on Human Factors in Computing Systems* (Denver, Colorado, USA, 2017).

9. Y. Huang, J. Zhou, P. Ke, X. Guo, C. K. Yiu, K. Yao, S. Cai, D. Li, Y. Zhou, J. Li, T. H. Wong, Y. Liu, L. Li, Y. Gao, X. Huang, H. Li, J. Li, B. Zhang, Z. Chen, H. Zheng, X. Yang, H. Gao,

Z. Zhao, X. Guo, E. Song, H. Wu, Z. Wang, Z. Xie, K. Zhu, X. Yu, A skin-integrated multimodal haptic interface for immersive tactile feedback. *Nat. Electron.* **6**, 1020–1031 (2023).

10. A. Crema, N. Malešević, I. Furfaro, F. Raschellà, A. Pedrocchi, S. Micera, A wearable multi-site system for NMES-based hand function restoration. *IEEE Trans. Neural Syst. Rehabil. Eng.* **26**, 428–440 (2018).

11. D. B. Popović, M. B. Popović, Automatic determination of the optimal shape of a surface electrode: Selective stimulation. *J. Neurosci. Methods* **178**, 174–181 (2009).

12. Y. Wang, L. Yin, Y. Bai, S. Liu, L. Wang, Y. Zhou, C. Hou, Z. Yang, H. Wu, J. Ma, Y. Shen, P. Deng, S. Zhang, T. Duan, Z. Li, J. Ren, L. Xiao, Z. Yin, N. Lu, Y. Huang, Electrically compensated, tattoo-like electrodes for epidermal electrophysiology at scale. *Sci. Adv.* **6**, eabd0996 (2020).

13. S. Yang, J. Cheng, J. Shang, C. Hang, J. Qi, L. Zhong, Q. Rao, L. He, C. Liu, L. Ding, M. Zhang, S. Chakrabarty, X. Jiang, Stretchable surface electromyography electrode array patch for tendon location and muscle injury prevention. *Nat. Commun.* **14**, 6494 (2023).

14. C. Wang, H. Wang, B. Wang, H. Miyata, Y. Wang, M. O. G. Nayeem, J. J. Kim, S. Lee, T. Yokota, H. Onodera, T. Someya, On-skin paintable biogel for long-term high-fidelity electroencephalogram recording. *Sci. Adv.* **8**, eabo1396 (2022).

15. K. Yang, C. Freeman, R. Torah, S. Beeby, J. Tudor, Screen printed fabric electrode array for wearable functional electrical stimulation. *Sens. Actuators A Phys.* **213**, 108–115 (2014).

16. H. Li, J. Cao, R. Wan, V. R. Feig, C. M. Tringides, J. Xu, H. Yuk, B. Lu, PEDOTs-based conductive hydrogels: Design, fabrications, and applications. *Adv. Mater.* **37**, e2415151 (2025).

17. S. Patel, F. Ershad, J. Lee, L. Chacon-Alberty, Y. Wang, M. A. Morales-Garza, A. Haces-Garcia, S. Jang, L. Gonzalez, L. Contreras, A. Agarwal, Z. Rao, G. Liu, I. R. Efimov, Y. S. Zhang, M.

Zhao, R. R. Isseroff, A. Karim, A. Elgalad, W. Zhu, X. Wu, C. Yu, Drawn-on-skin sensors from fully biocompatible inks toward high-quality electrophysiology. *Small* **18**, e2107099 (2022).

18. F. Ershad, A. Thukral, J. Yue, P. Comeaux, Y. Lu, H. Shim, K. Sim, N.-I. Kim, Z. Rao, R. Guevara, L. Contreras, F. Pan, Y. Zhang, Y.-S. Guan, P. Yang, X. Wang, P. Wang, X. Wu, C. Yu, Ultra-conformal drawn-on-skin electronics for multifunctional motion artifact-free sensing and point-of-care treatment. *Nat. Commun.* **11**, 3823 (2020).

19. F. Ershad, M. Houston, S. Patel, L. Contreras, B. Koirala, Y. Lu, Z. Rao, Y. Liu, N. Dias, A. Haces-Garcia, W. Zhu, Y. Zhang, C. Yu, Customizable, reconfigurable, and anatomically coordinated large-area, high-density electromyography from drawn-on-skin electrode arrays. *PNAS Nexus* **2**, pgac291 (2023).

20. Y. Ohm, C. Pan, M. J. Ford, X. Huang, J. Liao, C. Majidi, An electrically conductive silver–polyacrylamide–alginate hydrogel composite for soft electronics. *Nat. Electron.* **4**, 185–192 (2021).

21. M. I. Jordan, T. M. Mitchell, Machine learning: Trends, perspectives, and prospects. *Science* **349**, 255–260 (2015).

22. A. Moin, A. Zhou, A. Rahimi, A. Menon, S. Benatti, G. Alexandrov, S. Tamakloe, J. Ting, N. Yamamoto, Y. Khan, F. Burghardt, L. Benini, A. C. Arias, J. M. Rabaey, A wearable biosensing system with in-sensor adaptive machine learning for hand gesture recognition. *Nat. Electron.* **4**, 54–63 (2021).

23. J. Rafiee, M. A. Rafiee, F. Yavari, M. P. Schoen, Feature extraction of forearm EMG signals for prosthetics. *Expert Syst. Appl.* **38**, 4058–4067 (2011).

24. F. S. Botros, A. Phinyomark, E. J. Scheme, Electromyography-based gesture recognition: Is it time to change focus from the forearm to the wrist? *IEEE Trans. Industr. Inform.* **18**, 174–184 (2022).

25. J. Too, A. R. Abdullah, N. M. Saad, Classification of hand movements based on discrete wavelet transform and enhanced feature extraction. *Int. J. Adv. Comput. Sci. Appl.* **10**, 83–89 (2019).

26. A. Phinyomark, P. Phukpattaranont, C. Limsakul, Feature reduction and selection for EMG signal classification. *Expert Syst. Appl.* **39**, 7420–7431 (2012).

27. A. Phinyomark, P. Phukpattaranont, C. Limsakul, Fractal analysis features for weak and single-channel upper-limb EMG signals. *Expert Syst. Appl.* **39**, 11156–11163 (2012).

28. O. W. Samuel, H. Zhou, X. Li, H. Wang, H. Zhang, A. K. Sangaiah, G. Li, Pattern recognition of electromyography signals based on novel time domain features for amputees' limb motion classification. *Comput. Electr. Eng.* **67**, 646–655 (2018).

29. D. Tkach, H. Huang, T. A. Kuiken, Study of stability of time-domain features for electromyographic pattern recognition. *J. Neuroeng. Rehabil.* **7**, 21 (2010).

30. N. Ma, X. Zhang, H.-T. Zheng, J. Sun, in *Proceedings of the European conference on computer vision (ECCV)*. (Computer Vision Foundation, 2018), pp. 116–131.

31. F. Rattay, The basic mechanism for the electrical stimulation of the nervous system. *Neuroscience* **89**, 335–346 (1999).

32. D. R. McNeal, Analysis of a model for excitation of myelinated nerve. *IEEE Trans. Biomed. Eng.* **23**, 329–337 (1976).

33. J. D. Gomez-Tames, J. Gonzalez, W. Yu, in *2012 Annual International Conference of the IEEE Engineering in Medicine and Biology Society*. (2012), pp. 3576–3579.

34. J. Ladenbauer, K. Minassian, U. S. Hofstoetter, M. R. Dimitrijevic, F. Rattay, Stimulation of the human lumbar spinal cord with implanted and surface electrodes: A computer simulation study. *IEEE Trans. Neural Syst. Rehabil. Eng.* **18**, 637–645 (2010).

35. P. Li, J. Zhou, Y. Cui, J. Ouyang, Z. Su, Y. Zou, J. Liang, F. Wang, K. He, Y. Liu, Z. Zeng, F. Fang, C. Hou, N. Zhou, T. Peng, Q. Yuan, G. Tao, A scalable, robust and high-sensitivity fiber sensor for real-time body temperature monitoring. *Soft Sci.* **5**, 5–13 (2025).

36. A. Kuhn, T. Keller, M. Lawrence, M. Morari, The influence of electrode size on selectivity and comfort in transcutaneous electrical stimulation of the forearm. *IEEE Trans. Neural Syst. Rehabil. Eng.* **18**, 255–262 (2010).

37. A. Kuhn, T. Keller, S. Micera, M. Morari, Array electrode design for transcutaneous electrical stimulation: A simulation study. *Med. Eng. Phys.* **31**, 945–951 (2009).

38. Z. Lertmanorat, K. J. Gustafson, D. M. Durand, Electrode array for reversing the recruitment order of peripheral nerve stimulation: Experimental studies. *Ann. Biomed. Eng.* **34**, 152–160 (2006).

39. F. Rattay, Modeling the excitation of fibers under surface electrodes. *IEEE Trans. Biomed. Eng.* **35**, 199–202 (1988).

40. F. H. Netter, *Atlas of human anatomy, Professional Edition E-Book: Including NetterReference. com Access with full downloadable image Bank* (Elsevier health sciences, 2014).

41. C. Gabriel, S. Gabriel, E. Corthout, The dielectric properties of biological tissues: I. Literature survey. *Phys. Med. Biol.* **41**, 2231–2249 (1996).

42. A. Kuhn, T. Keller, M. Lawrence, M. Morari, A model for transcutaneous current stimulation: Simulations and experiments. *Med. Biol. Eng. Comput.* **47**, 279–289 (2009).

43. J. P. Reilly, *Applied Bioelectricity: From Electrical Stimulation to Electropathology* (Springer Science & Business Media, 2012).

44. S. Y. Chiu, J. M. Ritchie, R. B. Rogart, D. Stagg, A quantitative description of membrane currents in rabbit myelinated nerve. *J. Physiol.* **292**, 149–166 (1979).

45. J. Sweeney, J. Mortimer, D. Durand, in *IEEE 9th Annual Conference of the Engineering in Medicine and Biology Society*. (1987), vol. 3, pp. 1577–1578.

46. F. Rattay, Analysis of the electrical excitation of CNS neurons. *IEEE Trans. Biomed. Eng.* **45**, 766–772 (1998).
